# Developing quality fidelity and engagement measures for complex health interventions

**DOI:** 10.1111/bjhp.12394

**Published:** 2019-11-06

**Authors:** Holly Walton, Aimee Spector, Morgan Williamson, Ildiko Tombor, Susan Michie

**Affiliations:** ^1^ Department of Applied Health Research University College London UK; ^2^ Department of Clinical, Educational and Health Psychology University College London UK; ^3^ School of Social Sciences University of Westminster London UK; ^4^ Department of Behavioural Science and Health University College London UK

**Keywords:** complex health intervention, dementia, engagement, fidelity of delivery, implementation, measures, psychometric, quality

## Abstract

**Objectives:**

To understand whether interventions are effective, we need to know whether the interventions are delivered as planned (with fidelity) and engaged with. To measure fidelity and engagement effectively, high‐quality measures are needed. We outline a five‐step method which can be used to develop quality measures of fidelity and engagement for complex health interventions. We provide examples from a fidelity study conducted within an evaluation of an intervention aimed to increase independence in dementia.

**Methods:**

We propose five steps that can be systematically used to develop fidelity checklists for researchers, providers, and participants to measure fidelity and engagement. These steps include the following: (1) reviewing previous measures, (2) analysing intervention components and developing a framework outlining the content of the intervention, (3) developing fidelity checklists and coding guidelines, (4) obtaining feedback about the content and wording of checklists and guidelines, and (5) piloting and refining checklists and coding guidelines to assess and improve reliability.

**Results:**

Three fidelity checklists that can be used reliably were developed to measure fidelity of and engagement with, the Promoting Independence in Dementia (PRIDE) intervention. As these measures were designed to be used by researchers, providers, and participants, we developed two versions of the checklists: one for participants and one for researchers and providers.

**Conclusions:**

The five steps that we propose can be used to develop psychometrically robust and implementable measures of fidelity and engagement for complex health interventions that can be used by different target audiences. By considering quality when developing measures, we can be more confident in the interpretation of intervention outcomes drawn from fidelity and engagement studies.

**Statement of contribution**

***What is already known on the subject?***

- Fidelity and engagement can be measured using a range of methods, such as observation and self‐report.
- Studies seldom report psychometric and implementation qualities of fidelity measures.

***What does this study add?***

- A method for developing fidelity and engagement measures for complex health interventions.
- Guidance on how to consider quality when developing fidelity and engagement measures.

## Background

Measuring fidelity of delivery and engagement alongside the delivery of a trial helps us to understand whether planned interventions were effective (Borrelli, 2011). Fidelity of delivery is the extent to which interventions are delivered as planned (Borrelli, 2011). Consistent with previous research (Walton, Spector, Tombor, & Michie, 2017), engagement is used as an umbrella term to refer to whether a participant understands and can perform the required skills (receipt) and whether they can put plans into practice in daily life (enactment) (Borrelli, 2011). In this manuscript, we collectively refer to receipt and enactment as engagement to distinguish between provider behaviours (fidelity of delivery) and participant behaviours (engagement) (Walton *et al*., 2017). The definitions used in this article are based on the National Institutes of Health Behaviour Change Consortium framework for fidelity of delivery, intervention receipt, and intervention enactment (Bellg *et al*., 2004).

Without understanding whether interventions are delivered as planned and engaged with, it is difficult to fully understand whether or not an intervention is effective. Therefore, measuring fidelity and engagement as part of a process evaluation is essential for understanding how and whether an intervention works (Moore *et al*., 2015; Oakley, Strange, Bonell, Allen, & Stephenson, 2006). This is particularly important for complex interventions, which have many components.

Despite the importance of fidelity and engagement, fewer than half of the studies (24/66) included in a review of complex health behaviour change interventions measured both fidelity and engagement (Walton *et al*., 2017). To measure fidelity, observational, self‐report, and multiple measures have been used (Breitenstein *et al*., 2010; Lorencatto, West, Christopherson, & Michie, 2013; Toomey, Matthews, & Hurley, 2017; Walton *et al*., 2017). Audio‐recording all sessions and using multiple researchers to reliably rate a percentage for fidelity is the current gold standard (Lorencatto *et al*., 2013). To measure engagement, self‐report, attendance records, and multiple measures have been used (Gearing *et al*., 2011; Hankonen *et al*., 2015; Rixon *et al*., 2016; Walton *et al*., 2017). There is currently no consensus regarding the gold standard method to measure engagement in face‐to‐face interventions (Walton *et al*., 2017). Different aspects of engagement can be measured in different ways. For example, receipt has been measured most commonly using quantitative measures (Rixon *et al*., 2016). Enactment is difficult to measure as, researchers propose that in order to distinguish between outcomes and enactment, measures for enactment need to be specific to intervention skills rather than the target behaviour (Resnick *et al*., 2005). To overcome limitations of individual measures, multiple measures of fidelity and engagement are recommended (Keller‐Margulis, 2012; McKenna, Flower, & Ciullo, 2014; Munafo & Smith, 2018).

To ensure that fidelity measurements are trustworthy, psychometric and implementation qualities of measures should be reported (Walton *et al*., 2017). Psychometric qualities include the following: reliability (consistency of results in different situations; e.g., inter‐rater agreement) and validity (measures assessing what they aim to; e.g., sampling across different providers, sites, and time points) (Roberts, Priest, & Traynor, 2006; Walton *et al*., 2017). Implementation qualities include the following: acceptability of measures in relation to the needs of the intended audience (e.g., providers’ attitudes towards measurements) and practicality of the measures in relation to ease of completion and minimizing burden (e.g., availability of resources) (Lohr, 2002; Walton *et al*., 2017).

Despite the importance of high‐quality measures, considerations of quality are seldom reported in fidelity studies (Rixon *et al*., 2016; Walton *et al*., 2017). A review of fidelity and engagement measures used in complex health behaviour change interventions found that 74.2% of studies report at least one ‘psychometric quality’ (the quality of the measures) whereas only 25.8% report at least one ‘implementation quality’ (how the measures were used in practice) (Walton *et al*., 2017). This highlights the need to consider and report quality when measuring fidelity and engagement in complex health interventions. Consideration of these qualities is particularly pertinent in complex interventions, in which measuring fidelity and/or engagement may not be straightforward. For example, previous research found that agreement was difficult to achieve when measuring fidelity of the Community Occupational Therapy in Dementia‐UK (COTiD‐UK) intervention (Walton *et al*., submitted). To improve the quality of fidelity and engagement measures for complex interventions, guidance on how to develop high‐quality fidelity and engagement measures is needed.

To the authors’ knowledge, there is a lack of practical guidance on how to consider the quality of the measures and how they are used in practice when developing fidelity and engagement measures for complex health interventions. This manuscript builds on the findings from an earlier review (Walton *et al*., 2017) and provides recommendations on how to develop measures of fidelity and engagement for complex health interventions, with consideration around psychometric and implementation qualities. These measures of fidelity and engagement can be used by researchers, intervention providers (those that deliver the intervention to participants), and participants. This five‐step method will be illustrated using examples from the fidelity assessment conducted within an intervention aimed to increase independence in dementia (Promoting Independence in Dementia: PRIDE; See Csipke *et al*., 2018 and Box 1 for further details about PRIDE).

Box 1 **Description of the PRIDE intervention (Csipke *et al*., 2018)**

**PRIDE intervention**

- Aimed to improve independence for people living with mild dementia.
- Complex, tailored, manual‐based feasibility trial.
- Delivered by dementia advice workers (DAWs) – termed ‘providers’ in this manuscript (*n* = 12).
- Delivered to people living with mild dementia and their supporters (e.g., family members/friends) (*n* = 34) across four sites.
- Delivered over three sessions.
- Participants chose up to three tailored topics from a choice of seven topics: (1) keeping mentally active, (2) keeping physically active, (3) keeping socially active, (4) making decisions, (5) getting your message across, (6) receiving a diagnosis, (7) keeping healthy.
- Participants chose activities to work on, reviewed plans and identified barriers, facilitators and solutions.

In this fidelity assessment, a longitudinal observational design was used and fidelity was measured using observation (researcher ratings of transcribed, audio‐recorded intervention sessions) and provider and participant self‐report measures. Fidelity ratings from researchers, providers, and participants were compared. Engagement, including participants’ receipt (whether participants understood the information) and enactment (whether participants’ put their plans into practice between sessions), was measured using participant self‐report, which is consistent with previous research. Further details about the results of the fidelity assessment are reported elsewhere (see Walton, 2018).

This study is part of a larger mixed‐methods process evaluation which also included interviews with providers, participants, and supporters to qualitatively explore barriers and facilitators to fidelity and engagement and to develop recommendations to improve fidelity and engagement (see Walton, 2018).

## Methods

### Ethical approval

Ethical and research governance requirements were followed. Data were transcribed professionally and all transcripts were fully anonymized. Individuals were unidentifiable from data or resulting outputs. Ethical approval was obtained from the NHS East Midlands – Nottingham 1 Research Ethics committee (REC reference number: 16/EM/0044). Data were accessed by authorized study members and stored securely in a central location.

### Proposed methodology for developing fidelity measures

Fidelity checklists, which can be used to measure both fidelity and engagement, were iteratively developed using five steps. The process for applying these five steps to develop fidelity and engagement measures is outlined in Table 1.

**Table 1:** The five steps used to develop quality fidelity and engagement measures

Step	Proposed procedure	How to apply this step
1) Review previous measures	1a) Review measures used in fidelity assessments within your field and/or related fields	Look at other checklists used to measure fidelity in your field to see what have been included in their checklist and to help you make decisions about what to use in your own checklist (e.g., types of response options)
2) Analyse intervention components and develop an intervention framework	2a) Analyse intervention components	Read and code intervention materials (e.g., intervention manual) for key ‘components’ (aspects of an intervention that need to be delivered). To code intervention content, comment functions in word, and PDF can be used Consider what level of coding is appropriate (e.g., identifying components that should be delivered generally or identifying specific behaviour change techniques (Michie *et al*., 2013) that are present in the intervention) If intervention development occurs at the same time as this step, review the list of key components once the intervention/manual is updated and finalized
	2b) Group the list of components into categories	Identify similarities between your intervention components and develop these into groups. For example, at the start of the intervention, there may be lots of components used to deliver information – these may all be grouped under ‘Necessary basic information’
	2c) Develop a comprehensive intervention framework	Develop an intervention framework. This should be a table which provides an overview of what the intervention is, what components it includes, and other relevant aspects such as: who these components should be delivered to, and how these components link to the wider intervention aims and target behaviours The framework can be structured around your groups of components identified in 2b and could include columns with headings such as ‘Key targets’ (i.e., groups from 2c), intervention components, session number(s) that the component is delivered in, target behaviour that the component aims to improve, and intervention objectives
	2d) Remove redundant components from framework	Review the framework and identify and remove redundant components (e.g., components that have similarities with other components)
3) Develop fidelity checklists	3a) Identify which components from the framework take place in which of the intervention sessions	Use the column in your framework referring to session number to identify components for each of the intervention sessions
	3b) Develop one checklist for each of your intervention sessions, based on your framework	For each intervention session, use the intervention manual to put the components in the order that they will be delivered to participants Develop a checklist for each session which lists all the standardized components that should be delivered to all participants Choose which response options you would like to use (e.g., done, done to some extent, and not done) If the intervention has standardized (delivered to all) and tailored (participant choice) components, develop your fidelity checklist using the standardized components so that this can be compared across participants and providers and sites. Then, consider developing a separate table to supplement this checklist to capture delivery of the tailored components. One way could be to develop a separate tailored grid to explore one of the standardized components in more detail If measuring fidelity and engagement, checklists can also include questions on what participants understood and put into practice
	3c) Tailor checklists for use by your intended audiences	Consider creating different versions of the fidelity checklists for different audiences (e.g., researchers, providers, and participants) Different wording may be appropriate for different audiences, for example, provider and researcher checklists may be written in terms of delivery, whereas participant checklists may be written in terms of what is received
	3d) Review the checklists	Review the checklists to identify and remove redundant components (e.g., repetition) or jargon
	3e) Develop simple guidelines for all target users which explain how to complete the checklists	Develop guidelines for all intended users of the checklists (researchers, providers, and participants) Guidelines will serve different purposes for different audiences and therefore may include different content ○ Researcher guidelines could include simple but in‐depth: (1) information about the checklists, (2) guidance on how to complete the checklists, (3) guidance on how to decide which score to give, (4) definitions for each component, (5) illustrative examples for ‘done’, ‘done to some extent’, and ‘not done’ for all components across all sessions ○ Provider guidelines could include simple explanations of (1) what the checklists and audio‐recordings are for, (2) what to return and how, (3) how to fill out the checklists, (4) an example checklist ○ Participant guidelines could include a simple explanation on: (1) how to complete the forms, (2) how to return the forms, (3) an example checklist
4) Obtain feedback about the content and wording of the checklists and guidelines	4a) Ask relevant stakeholders to give feedback on the content and wording of checklists and coding guidelines	Decide who you need to get feedback from. This could include intervention team members with expertise of developing the intervention and/or target users of the checklists Ask stakeholders to provide feedback on the content and wording of checklists and coding guidelines.
	4b) Edit checklists and guidelines to take this feedback into account	In line with feedback received, make changes to the checklists and guidelines Consider consulting relevant guidance (e.g., condition‐specific guidance) and/or readability statistics to make sure that checklists are as easy to use as possible
5) Pilot and refine checklists and coding guidelines to assess and improve reliability *Note. This step is only necessary if researchers will be carrying out the fidelity assessment (i.e., not if only using provider/participant self‐* *report)*	5a) Use multiple researchers to test coding guidelines and checklists against some initial intervention transcripts (initial piloting)	Decide on a reliability threshold that you use to determine reliability of researcher coding (e.g., weighted kappa, kappa or percentage agreement) Select a percentage of your overall fidelity sample to pilot (e.g., 10% of your overall sample) Transcribe these sessions Identify a second researcher Together with the second researcher, independently ‘code’ the transcripts discussion. Coding the transcript means applying the coding guideline to the transcript, and identifying evidence for the delivery each component within that transcript To ‘code’ a transcript, use the comment function in Microsoft word to identify evidence for each component. Add a comment with the number of the component and a note to say whether the component was delivered/partly delivered/not delivered Add explanations for coding choices, where necessary to facilitate discussions Each researcher should complete a checklist for each of the sessions that they rate. Save the checklists with the researcher's initials
	5b) Discuss discrepancies and amend coding guidelines	Work out agreement between the two researchers using your chosen reliability statistic and identify components that have been disagreed on (e.g., by creating a spreadsheet to record these disagreements) Meet to discuss coding and discrepancies. To do this, go through each component that was disagreed on and outline reasons for the score that you gave and use the coded word document to support this discussion. Decide between you which of the scores is the most appropriate and agree on this Note down the reasons for disagreement between coders Create a third version of the checklists to record final agreed scores on. For clarity, save this checklist with ‘agreed’ and both of your initials at the end of the file name Identify and record reasons for discrepancies Amend coding guidelines where necessary to improve clarity
	5c) Pilot and amend coding guidelines until selected agreement threshold is achieved	Repeat stages 5a and 5b until chosen reliability threshold is achieved Note. More sets of sessions may need to be transcribed and included in your pilot to achieve agreement If coding guidelines are amended throughout the piloting process, the transcripts used for piloting may need to be re‐coded during your main fidelity assessment once coding guidelines have been finalized

Below, we briefly outline how these five steps were applied to develop fidelity and engagement measures for the PRIDE intervention. Due to time constraints associated with using these checklists in the feasibility trial, these checklists were iteratively developed alongside the intervention manual.

### Step 1: Review previous measures

After developing the PRIDE fidelity checklists, many of these steps were also followed to develop fidelity checklists for use in another complex intervention for people with dementia: The Community Occupational Therapy in Dementia – UK intervention (COTiD‐UK; see Walton *et al*., submitted). The fidelity checklists for PRIDE were developed prior to the development of COTiD‐UK checklists, but the fidelity assessment for COTiD‐UK took place at the same time as PRIDE.

Prior to the development of the PRIDE checklists, we were not aware of any fidelity checklists that had been used in similar dementia interventions. Instead, to inform the development of our checklists, we reviewed fidelity measures that were known to our team: checklists used in the Prediction and Management of Cardiovascular Risk for people with severe mental illnesses (PRIMROSE) project (Osborn *et al*., 2016).

### Step 2: Analyse intervention components and develop a framework outlining the content of the intervention

The framework described in this step is separate from the process of PRIDE intervention development (see Yates *et al*., 2019). The framework described in this manuscript should instead be considered as a tool to facilitate the development of fidelity checklists by clearly outlining the intervention content. To ensure that the intervention content matched the fidelity checklist content, we developed this framework from the intervention manual that was developed by the PRIDE intervention team. This framework was used to facilitate understanding of the PRIDE intervention manual and what should be delivered by providers.

#### 2a) Analyse intervention components

The PRIDE intervention manual was read and coded. This coding was used to identify key components of PRIDE (i.e., aspects of the intervention that need to be delivered to participants). We used the Behaviour Change Technique (BCT) Taxonomy Version 1 to identify BCTs (Michie *et al*., 2013).

#### 2b) Group the list of components into categories

Components were grouped into three categories by identifying similarities across components: necessary basic information, tailoring and assessment and PRIDE activities.

#### 2c) Develop a comprehensive intervention framework

Categories were used to develop an intervention framework which included the following: (1) key targets of the intervention, (2) key intervention components, (3) PRIDE session number that the component is delivered in, (4) target behaviour, (5) BCTs, and (6) PRIDE objectives (see Appendix S1 for the PRIDE intervention framework).

#### 2d) Review the framework

A team of behavioural scientists (the first, fourth, and last author) reviewed the intervention framework and removed redundant components.

### Step 3: Develop fidelity checklists

#### 3a) Identify which components from the framework take place in which of the intervention sessions

The PRIDE intervention framework was used to identify key components which should be delivered for each of the three PRIDE sessions.

#### 3b) Develop one checklist for each of the intervention sessions, based on the intervention framework

Three PRIDE fidelity checklists were developed (one for each session). These checklists all contained standardized components which all participants should receive. Components were put in order of delivery. Intervention components were worded in everyday appointment activities rather than BCTs so that delivery of components could be measured by all intended audiences (researchers, providers, and participants).

If components were tailored to participants’ individual choices (e.g., providing relevant resources), these were referred to as the ‘chosen topic’ in the checklist. To identify which tailored components were delivered for participants’ chosen topics, an additional grid was included in the researcher and provider checklists.

Participant checklists also contained questions on whether participants understood the information, knew how to put their plan into action, and practised and used these skills between sessions. Questions were developed based on the definitions of ‘receipt’ and ‘enactment’ (Bellg *et al*., 2004; Borrelli, 2011). Engagement questions were not included on the researcher/provider checklists as providers and researchers would be unable to answer questions on participants’ understanding. Similarly, providers and researchers were not present between sessions when enactment of plans would take place.

#### 3c) Tailor the checklists for use by the intended audiences

Two versions of these checklists were developed: one for providers and researchers, and one for participants (people living with dementia). Checklists were tailored and worded for the target audience. One checklist was developed to be used by both providers and researchers as the wording of the checklists applied to both groups. Participant checklists were worded in relation to receipt and provider checklists were worded in relation to delivery. For the provider checklists, we added a ‘brief reason’ column for them to add notes to explain why components were not delivered/partially delivered.

#### 3d) Review the checklists

The team of behavioural scientists reviewed the checklists to identify and remove redundant components and jargon.

#### 3e) Develop simple coding guidelines for all target users which explain how to complete the checklists

Simple guidelines were developed for all intended users (researchers, providers, and participants). The guidelines explained how to complete these checklists. In‐depth coding guidelines were developed for researchers (see Appendix S2). Researcher coding guidelines included definitions for each component and illustrative examples of ‘done’, ‘done to some extent’, and ‘not done’. Simple guidelines for providers and participants were also developed. Provider and participant guidelines provided information on what the checklists are for, how to complete and return the checklists, and an example checklist.

### Step 4: Obtain feedback about the content and wording of the checklists and guidelines from relevant stakeholders

#### 4a) Ask relevant stakeholders to give feedback on the content and wording of checklists and coding guidelines

This step ensured that the checklist and guideline items were relevant, accurate, and worded appropriately for use by providers and people living with dementia. Six members of the intervention development team provided feedback. We also asked for feedback from the intervention's Public Patient Involvement (PPI) group, providers, and a person living with dementia working in a PPI type role.

#### 4b) Edit checklists and coding guidelines to take feedback into account

Feedback from the intervention team and PPI group was used to refine the checklists. To enhance accessibility of the checklists for people living with dementia, condition‐specific guidance was used (Dementia Empowerment and Engagement Project; DEEP Guide, 2013). To determine whether checklists were easy to read, Flesch readability statistics (Flesch, 1948) were reviewed following feedback.

### Step 5: Pilot and refine checklists and coding guidelines to assess and improve reliability of researcher ratings

#### 5a) Use multiple researchers to test coding guidelines and checklists against some intervention transcripts (initial piloting)

To test coding guidelines and pilot the coding task, two researchers (independent researcher and 1st author) transcribed and coded an initial set of three transcripts (Session one, Session two and Session three).

#### 5b) Discuss discrepancies and amend coding guidelines

Discrepancies between coders were identified. Reliability was calculated using Cohen's weighted kappa and percentage agreement (Cohen, 1968; Gwet, 2014). Feedback from this process was used to amend the coding guidelines.

#### 5c) Pilot and amend coding guidelines until selected agreement threshold is achieved

After initial piloting, 17 further sets of transcripts were coded independently by two researchers (1st and 3rd author) until good agreement was achieved. To ensure that instructions were clear, coders discussed guidelines before coding. Missing responses were clarified with the coder prior to agreement calculations where possible. If responses were not clarified, these were included as missing responses.

Agreement was measured using Cohen's weighted kappa and percentage agreement (Cohen, 1968; Gwet, 2014). For standardized components, agreement was assessed using weighted kappa. For tailored components and individual topics, agreement was assessed using percentage agreement. To account for the ordinal nature of data and partial agreements, we used weighted kappa (Gwet, 2014). For example, a disagreement of ‘done’ and ‘done to some extent’ would be more of a partial agreement than one of ‘done’ and ‘not done’. Linear weights (agreements = 1.0, partial agreements = 0.5, disagreements = 0.0) were selected instead of quadratic weights. Linear weights were chosen as they provide equal spacing between options and do not overestimate reliability as much as quadratic weights (Gwet, 2014).

Higher kappa scores indicate better agreement (<0.00 is poor, 0–0.2 is slight, 0.21–0.40 is fair, 0.41–0.60 is moderate, 0.61–0.80 is good and 0.81–1 is excellent agreement; Gisev, Bell, & Chen, 2013; Landis & Koch, 1977; Viera & Garrett, 2005). To ensure high agreement, a threshold of >.60 kappa (good) was selected. A threshold of >.60 kappa (good) was selected and deemed to be appropriate. This is because kappa is a conservative estimate of reliability which ensures that chance agreements are accounted for (Lombard, Snyder‐Duch, & Bracken, 2002). To ensure that a high level of agreement was achieved and maintained, coders needed to achieve κ > 0.61 for three consecutive transcripts per session. This threshold was also consistent with the level of agreement used due to difficulties achieving excellent agreement within a fidelity evaluation of the COTiD‐UK intervention (Walton *et al*., submitted).

Coding guidelines were finalized once no further changes were necessary. We then re‐applied these finalized coding guidelines to all intervention transcripts to measure fidelity of, and engagement with PRIDE (not reported here, see Walton, 2018; Walton *et al*., in preparation).

## Results

### Development of fidelity checklists

**Figure 1:**
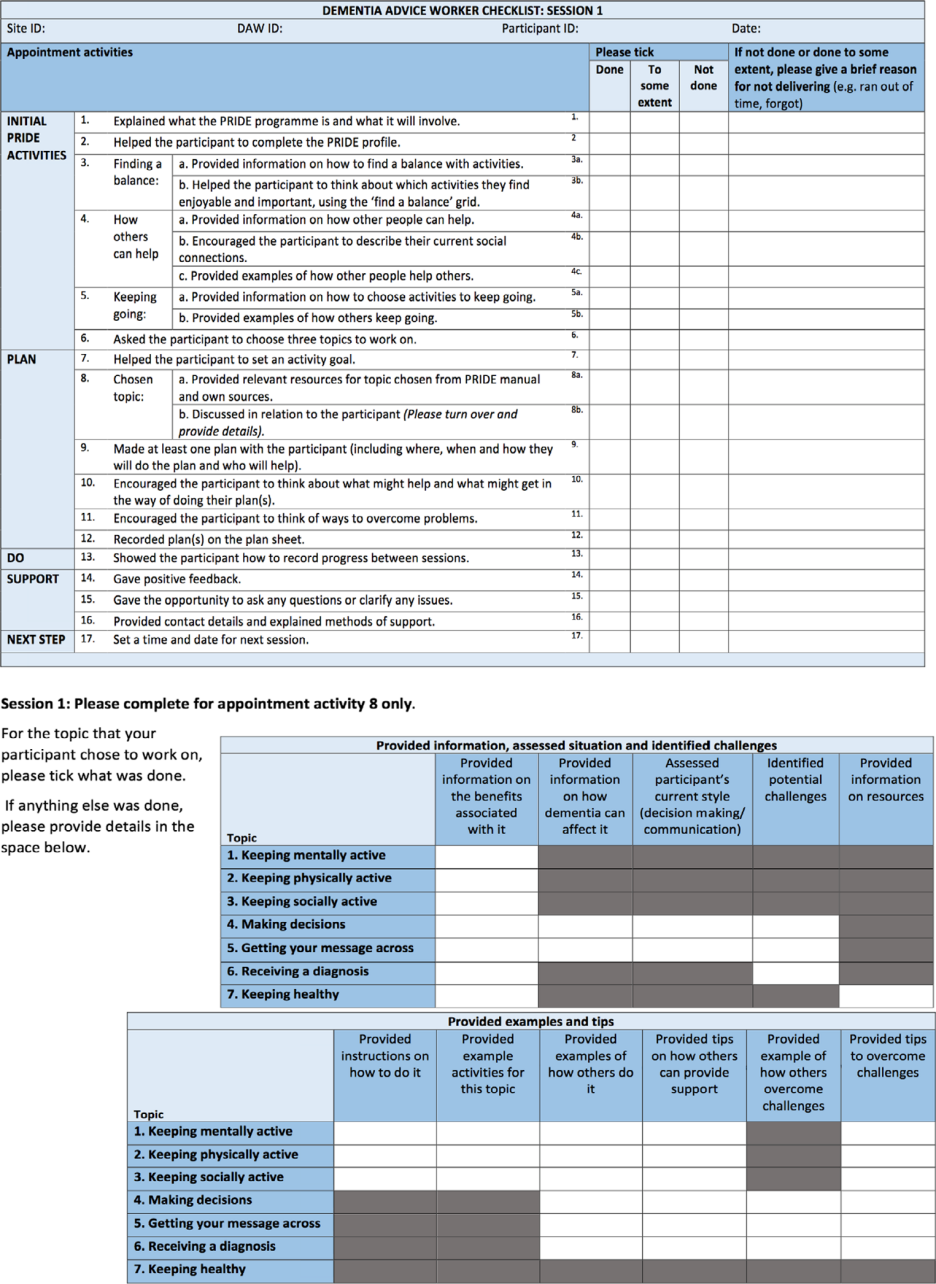
An example provider checklist (Session one). [Colour figure can be viewed at http://www.wileyonlinelibrary.com]

**Figure 2:**
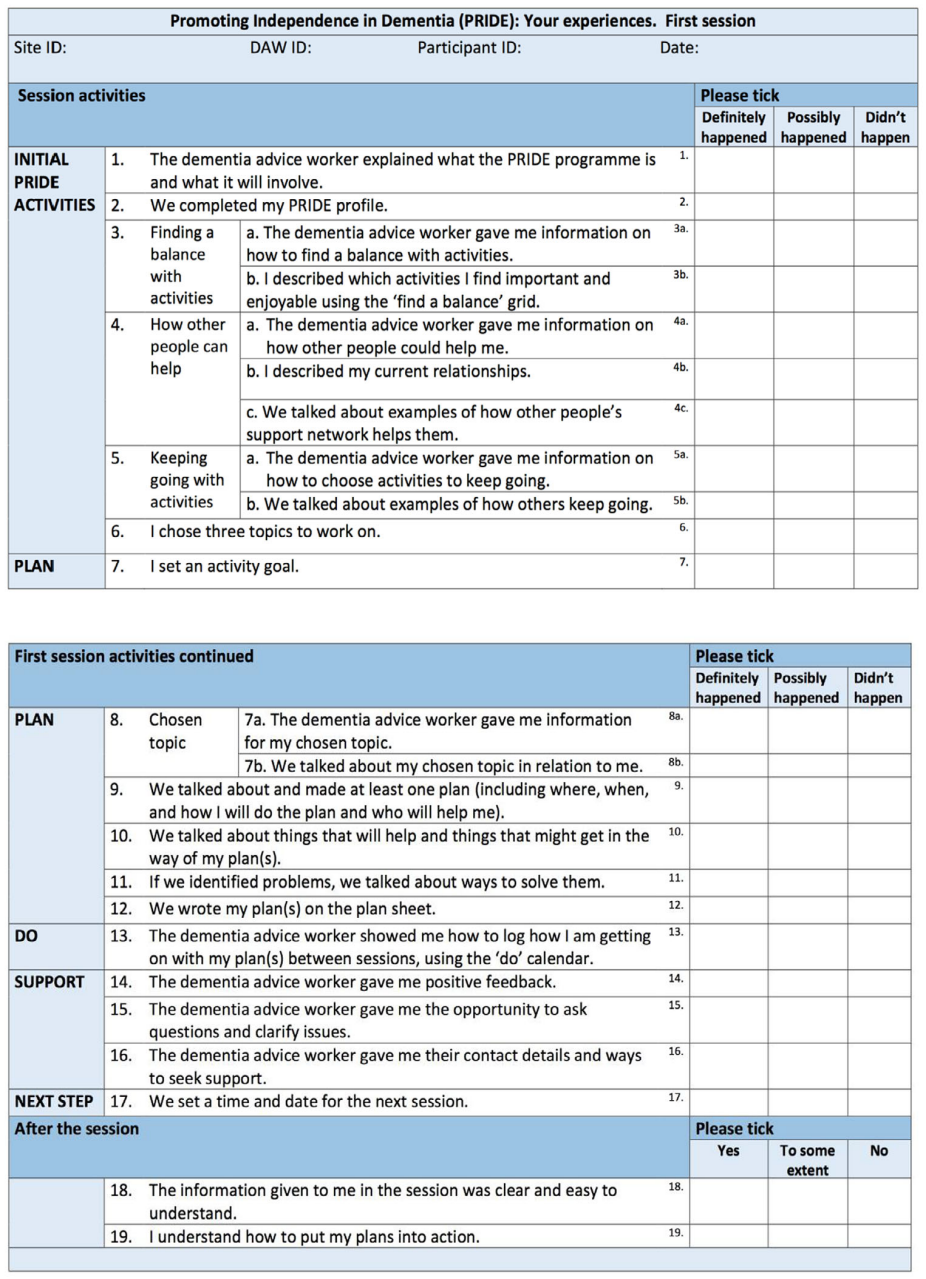
An example participant ‘your experience’ checklist (Session one). [Colour figure can be viewed at http://www.wileyonlinelibrary.com]

We developed three fidelity checklists (Session one, Session two, and Session three), each containing standardized intervention components (Session one: *n* = 22, Session two: *n* = 18, Session three: *n* = 12). Provider checklists also contained an additional grid for tailored components. See Figure 1 for an example of the provider and researcher checklists and Figure 2 for an example of the participant ‘your experience checklists’ (See Appendices S3 and S4 for full copies of both sets of checklists).

In the provider/researcher checklists, three response options were offered: ‘done’, ‘done to some extent’, and ‘not done’. A ‘reason’ column was added to the PRIDE provider checklists so that providers could add details or context to indicate a reason for why a component was not delivered**.** In the participant checklists, three response options were available for the questions on fidelity: ‘definitely happened’, ‘possibly happened’ and ‘didn't happen’, and three response options were available for the questions on engagement: ‘yes’, ‘to some extent’, and ‘no’.

Based on feedback, we amended the checklists to minimize jargon (e.g., replacing ‘facilitators’ with ‘things that will help with my plan’). To enhance accessibility of the checklists for people with dementia, the Dementia Empowerment and Engagement Project (DEEP) guidance (DEEP Guide, 2013) was used. This included enlarging the font size to 16pt, using a clear font style, using colour, avoiding jargon and academic terminology, removing passive voice, and explaining terms.

Finalized participant checklists had a Flesch score of 71.4 and a grade of 6.2, and the provider checklists had a Flesch score of 62.0 and a grade of 7.5. This is within the recommendations for the general population (Vahabi & Ferris, 1995); thus, the readability of the checklists was suitable.

### Response rates

To indicate how acceptable and practical the checklists were for use by providers and people with dementia, response rates were calculated. Ninety‐three sessions were delivered. Of these, 72 audio‐recordings (77.4%), 75 provider checklists (80.7%), and 59 participant checklists (63.4%) were returned. Reasons for not returning the audio‐recordings were as follows: technical failures during or after recording, the audio‐recording being wiped from the device before uploading, or a corrupt file. Of the 24 sets (*n* = 72 transcripts) sampled for the fidelity assessment, 17 recordings were missing, resulting in 55 transcripts.

For the researcher ratings, no components were missing, 13 components were scored ‘not applicable’, and no responses were unclear. Across the provider checklists, 30 individual components were missing, 11 components were scored ‘not applicable’ and one component was ‘unclear’. Across the participant checklists, 20 individual fidelity components were missing, one fidelity component was scored ‘not applicable’, and six fidelity components and two engagement components were ‘unclear’. ‘Missing’ components refer to components which participants did not complete a rating for.

### Inter‐rater agreement for researcher ratings (piloting stage)

For the standardized components, good inter‐rater agreement (κ > .61) was achieved after coding 12 Session one transcripts (κ = 0.8–0.9), 14 Session two transcripts (κ = 0.7–0.8), and 14 Session three transcripts (κ = 0.6–1.00) (initial pilot coding not included) (See Table 2). For Session one, inter‐rater agreement of >. 61 kappa was not achieved three times in a row due to an unequal distribution of responses (Feinstein & Cicchetti, 1990), which meant that kappa was moderate (κ = 0.4) but percentage agreement was very high (86.4%).

**Table 2:** Weighted kappa and percentage agreement for standardized components across PRIDE Sessions one, two, and three in both the piloting stage and main assessment stage

Set of transcripts		Weighted kappa (%)		
		Session 1	Session 2	Session 3
Piloting coding guidelines and checklists to achieve agreement				
1	Coding pair 1 (pilot)	0.21 (59.1)	0.26 (55.6)	−0.33 (50)
	Coding pair 2	0.38 (54.6)	0.4 (66.66)	−0.11 (66.66)
3		−0.2 (36.4)	0.48 (61.1)	−0.25 (41.66)
4		0.47 (63.6)	0.65 (72.2)^a^	0.49 (66.66)
5		0.55 (59.1)	0.62 (77.7)^a^	0.29 (58.33)
6		0.62 (77.3)^a^	0.69 (77.7)^a^	0.31 (50)
7		0.28 (68.2)	0.16 (50)	0.59 (66.6)
11^b^		0.56 (77.3)	0.54 (66.7)	0.00 (33.3)
2		0.83 (90.9)^a^	0.71 (77.7)^a^	No session
8		No transcript	No transcript	0.31 (58.3)
9		0.07 (72.7)	0.41 (61.1)	No transcript
10		0.85 (90.9)^a^	0.83 (83.3)^a^	No transcript
13		0.81 (86.4)^a^	No transcript	0.61 (66.66)^a^
12		No transcript	0.45 (55.6)	0.46 (58.33)
14		0.42 (86.4)^a^,^c^	No transcript	0.57 (66.66)
15		No transcript	No transcript	1.00 (100)^a^
16		No transcript	No transcript	0.68 (75)^a^
17		–	0.83 (83.3)^a^	No transcript
18		–	0.77 (83.3)^a^	0.64 (83.33)^a^
1 (re‐coded new guidelines)		–	0.68 (88.9)^a^	–
Main fidelity assessment				
5 (*)		0.7 (72.7)	0.5 (72.2)	0.3 (66.7)
6		–	0.4 (66.7)	0.8 (83.3)
7		–	0.4 (72.2)	–
18 (*Session 1)		0.4 (68.2)	Pre‐coded	Pre‐coded
19 (*Session 2)		0.6 (77.3)	0.5 (66.7)	0.4 (50)
20		0.8 (90.9)	0.7 (77.7)	0.6 (75)
23 (*)		0.8 (90.9)	0.5 (55.5)	No transcript
24		–	0.7 (83.3)	0.8 (91.7)

Notes

This was used when agreement had already been reached, and no further sessions needed to be coded until the next sampled set.

No transcript – refers to sessions where transcripts were not available to code.

(*) Sets in the main fidelity assessment that were selected for double coding.

Pre‐coded refers to sets that were coded during the piloting phase.

a Indicates agreement >0.61 was reached.

b Coding guidelines not changed after coding this set.

c Weighted kappa did not reach >0.61 however >85% agreement achieved three times in a row and >0.8 kappa 3 times in last five sets. Kappa low due to lots of ‘not done’ responses, despite only three disagreements.

Table 3 reports percentage agreement for tailored topics and components. Good agreement (average means: 54.6–87.8%) was achieved for tailored components in both sessions.

**Table 3:** Percentage agreement for delivery of tailored topics and topic components (scored out of 11) in PRIDE Sessions one and two in both the piloting stage and main assessment stage

Topic (number of sets delivered in Session 1 and 2)	Mean number of components agreed on (range) (%)	
	Session 1	Session 2
Piloting coding guidelines and checklists to achieve agreement		
Keeping mentally active (S1: 9, S2: 2)	75.7 (54.6–90.9)	86.4 (81.8–90.9)
Keeping physically active (S1: 3, S2: 0)	84.8 (72.7–90.9)	N/A
Keeping socially active (S1: 4, S2: 3)	86.4 (72.7–90.9)	87.8 (81.8–90.9)
Making decisions (S1: 2, S2: 1)	86.4 (81.8–90.9)	81.8
Getting your message across (S1: 4, S2: 1)	75 (27.3–90.9)	81.8
Receiving a diagnosis (S1: 1, S2: 2)	54.6	72.7 (63.6–81.8)
Keeping healthy (S1: 0, S2: 0)	N/A	81.8 (63.6–90.9)
No topics delivered (S1: 2, S2: 3)	N/A	N/A
Main fidelity assessment		
1 Keeping mentally active (S1: 4, S2: 1)	93.6 (81.8–100)	100
2 Keeping physically active (S1: 2, S2: 2)	90.9	90.9
3 Keeping socially active (S1: 2, S2: 2)	90.9 (81.8–100)	86.4 (81.8–90.9)
4 Making decisions (S1: 2, S2: 1)	81.8	63.6
5 Getting your message across (S1: 2, S2: 1)	81.8 (72.7–90.9)	81.8
6 Receiving a diagnosis (S1: 2, S2: 0)	95.5 (90.9–100)	N/A
7 Keeping healthy (S1: 0, S2: 2)	N/A	77.3 (63.6–90.9)
No topic delivered (S1: 0, S2: 3)	N/A	N/A

Note

N/A = not applicable: Topic not delivered.

11 components = 100%.

## Discussion

### Key findings

We have developed a systematic method consisting of five steps that can be used to develop measures of fidelity and engagement that consider both psychometric and implementation qualities. These measures can be used by different audiences including providers, participants, and researchers. The consideration of quality when developing fidelity and engagement measurements for the PRIDE intervention provides confidence in fidelity and engagement results obtained using these measures.

### Findings in relation to previous research

Findings from these studies extend previous work in this area by demonstrating that researchers can use these five steps to consider reliability, validity, practicality, and acceptability when developing measures of fidelity and engagement. These psychometric and implementation qualities have been recommended (Gearing *et al*., 2011; Glasgow *et al*., 2005; Holmbeck & Devine, 2009; Lohr, 2002; Stufflebeam, 2000), yet reported infrequently (Walton *et al*., 2017). These qualities were considered when developing PRIDE checklists.

To improve the consistency of fidelity coding, the checklists and coding guidelines were piloted until good inter‐rater agreement was achieved (Lorencatto, West, Bruguera, & Michie, 2014). The finding that good agreement was difficult to achieve highlights that while it is possible to achieve reliability, piloting checklists and coding guidelines is a necessary step when developing fidelity and engagement measures. This finding is consistent with previous fidelity research (Harting, van Assema, van der Molen, Ambergen, & de Vries, 2004; Thyrian *et al*., 2010; Walton *et al*., submitted). This may be due to the complexity of the intervention, which has been suggested to make it harder to achieve good agreement (Harting *et al*., 2004). To enhance agreement, clear definitions of components were provided in the researcher coding guidelines to make coding easier and limit individual judgement and subjectivity, as recommended by previous research (French *et al*., 2015; Hardeman *et al*., 2008; Harting *et al*., 2004; Keith, Hopp, Subramanian, Wiitala, & Lowery, 2010; Lorencatto *et al*., 2014).

The development of fidelity measures for use by multiple people (researchers, providers, and participants) contributes towards validity by ensuring that findings can be triangulated and that individual limitations are overcome by multiple measurements (Keller‐Margulis, 2012; McKenna *et al*., 2014; Munafo & Smith, 2018. In the PRIDE fidelity assessment, we found discrepancies between fidelity ratings, with researcher ratings indicating moderate fidelity and provider and participant ratings indicating high fidelity (see Walton *et al*., [Link]; Walton, 2018 for more details). In this study, the differences in measurement tools may lead to differences in fidelity ratings, as researchers had thorough coding guidelines to base their decisions on whereas providers and participants received simple guidelines to base their decisions on. Providing more thorough guidelines to providers and participants would have increased the time taken to complete checklists and complexity of the task, therefore this would not have been acceptable or practical to implement in this study.

This method highlights strategies that can be taken to enhance acceptability and practicality when developing measures of fidelity and engagement. To enhance acceptability and practicality, different versions of the checklists were created in the PRIDE study for different audiences (Glasgow *et al*., 2005; Holmbeck & Devine, 2009; Lohr, 2002). Providing a ‘reason’ column in the provider checklist aimed to provide an expectation that it is acceptable to not deliver all components, which may have enhanced acceptability. Feedback was sought on the content and wording of these checklists from PPI members and interventionists. This feedback, together with condition‐specific guidance (The Dementia Engagement and Empowerment Project (DEEP Guide), 2013), informed adaptations to improve ease of use and acceptability for participants and providers. Simple guidelines were developed to help participants and providers to try to enhance practicality (Lohr, 2002; Walton *et al*., 2017). While acceptability and practicality were not formally assessed, high response rates for audio‐recordings, participant and provider checklists offer an indication of acceptability and practicality (Walton, 2018).

### Limitations

Although feedback was sought from the fidelity and intervention development teams, only one researcher coded the intervention content and developed the framework of intervention components. Although BCTs (Michie *et al*., 2013) were highlighted from the PRIDE manual, these were used to develop an intervention framework but not the checklist components. Therefore, components in the checklists were not specifically measured using BCTs. Using everyday language to describe components enabled the PRIDE checklists to be accessible for all audiences, including providers and people with dementia.

A further limitation of the checklist development process was that only one previous measure of fidelity was formally reviewed in step 1 of checklist development. Future research should consider reviewing a wider range of fidelity checklists prior to steps 2–5.

While we gained feedback on the checklists from the PRIDE PPI group, we only received feedback on the checklist wording from one person living with dementia. However, alongside this feedback, we also reviewed guidance which was co‐produced with people living with dementia (DEEP, 2013), to ensure that checklists were as accessible as possible for people living with dementia to use.

One limitation of this study is that we used participant self‐report to measure engagement (receipt and engagement). Objective measures of participant engagement may have helped to overcome limitations of self‐report such as social desirability bias. Furthermore, participants were asked to complete checklists as soon as possible after each session but in some cases this may not have happened. Therefore, there may have been some difficulties for participants remembering the extent to which they engaged or the extent to which the intervention was delivered as planned. Asking participants to complete a couple of extra questions was practical as participants only had to complete one measure which included both fidelity and engagement. We also triangulated findings with more in‐depth qualitative findings on barriers and facilitators to engagement from perspectives of participants and supporters. These findings are reported along with the engagement outcomes to develop recommendations for improving engagement (see Walton *et al*., in preparation).

This study only focused on fidelity and engagement and did not develop measures to evaluate therapeutic alliance or the relationship between the patient and provider. However, the relationship between participants and providers was explored when conducting interviews to identify barriers and facilitators to engagement, as part of the wider project.

### Implications

These five steps can inform the development of quality fidelity and engagement measures that can be implemented by researchers, providers, and participants for complex health interventions for different populations and is not limited to dementia interventions. Developing high‐quality measures with good psychometric and implementation qualities can advance our understanding of fidelity and engagement outcomes and help us interpret intervention effectiveness more accurately.

The checklists developed from these five steps can be used to measure fidelity of delivery and engagement. Findings from fidelity and engagement assessments can help researchers to understand which components of an intervention were not delivered. From this, difficult to deliver components can be identified and together with interviews exploring barriers and facilitators to delivery; recommendations to improve fidelity of delivery, and training for providers can be developed. Similarly, by understanding participants’ levels of engagement with an intervention, recommendations to improve engagement can be developed.

### Future research

Future research could consider how best to formally measure validity, acceptability, and practicality of fidelity and engagement measures. This would help to determine whether measures are in fact high‐quality.

The development of these checklists was part of a larger process evaluation of PRIDE, in which we assessed fidelity and engagement and qualitatively explored barriers and facilitators to fidelity of delivery and engagement (Walton, 2018). These findings will be used to develop recommendations to improve fidelity of delivery and engagement.

### Conclusions

Researchers can follow these five steps to develop psychometrically robust and implementable fidelity and engagement measures for complex health interventions that can be used by different audiences, including researchers, providers, and participants. By considering quality when developing measures, we can be more confident in the interpretation of intervention outcomes drawn from fidelity and engagement studies.

The checklists developed in this study were used to measure fidelity of delivery of, and engagement with PRIDE. Together with findings from a qualitative exploration of fidelity and engagement, the findings from fidelity assessments can be used to develop recommendations to improve fidelity of delivery and engagement.

## Compliance with ethical standards

The PRIDE programme of research received Health Research Authority approval and NHS ethical approval as part of the feasibility trials intervention ethics application (NHS East Midlands–Nottingham 1 Research Ethics Committee, REC reference number: 16/EM/0044).

## Conflicts of interest

The authors declare no conflicts of interest.

## Supporting information


**Appendix S1.** PRIDE Intervention framework.
**Appendix S2.** PRIDE coding guidelines for researchers.
**Appendix S3.** Provider/researcher fidelity checklists, Sessions 1–3.
**Appendix S4.** Participant ‘your experience’ fidelity checklists, Sessions 1–3.

## Data Availability

The data that support the findings of this study are available from the corresponding author upon reasonable request.
