# Supplementary material for: Developing quality fidelity and engagement measures for complex health interventions
Source: Br J Health Psychol. 2019 Nov 6;25(1):39–60. doi: 10.1111/bjhp.12394 (PMC7004004; doi:10.1111/bjhp.12394)

Appendix 1 – PRIDE Intervention framework

The PRIDE intervention aims to promote independence and facilitate the person’s access to opportunities which will help them live well with dementia. It also aims to enable the participants to maintain: an active lifestyle, a healthy lifestyle and maintain cognitive activities and social roles. Therefore, the components of the intervention which are most relevant to these objectives (key components) have been identified.

| **Framework** | | **Key targets** | **Key components*** | **Session** | **Target behaviour** | **BCT’s** | **PRIDE objectives** |
| --- | --- | --- | --- | --- | --- | --- | --- |
| Necessary information | 1. Introduction to research | | Purpose of PRIDE intervention was explained   - What it is - What it involves - What the purpose is - How the manual works - Participant choice | 1 | N/A | N/A | N/A |
|  | 1. Information provision | | 1. Information and tips on how to find a balance | 1 | Carrying out activities, Participating in activities, Developing a routine | 1.1 Goal setting behaviour  3.1 Social support unspecified  3.2 Practical social support  4.1 Instruction on how to perform the behaviour  7.1 Prompts/cues  8.1 Behavioural practice/rehearsal   - 1. Habit formation | Independence, Active lifestyle, Healthy lifestyle, Cognitive activities |
|  |  |  | 1. Information and tips on social connections | 1 | Connecting with others, Support for activities, Communicating with healthcare professionals | 1.2 Problem solving  3.1 Social support unspecified  4.1 Instruction on how to perform the behaviour  5.3 Information about social and environmental consequences  6.1 Instruction on how to perform the behaviour | Social roles |
|  |  |  | 1. Information and tips on keeping going | 1 | Choosing activity, Planning activities, Being motivated | 3.1 Social support unspecified   - 1. Instruction on how to perform the behaviour | Active lifestyle, Healthy lifestyle |
|  | 1. Provide tailored advice** | | 1. Keeping mentally active | 1,2,3 | Carrying out activities to keep mentally active | 1.2 Problem solving  5.3 Information about social and environmental consequences   - 1. Graded tasks | Active lifestyle, Healthy lifestyle |
|  |  |  | 1. Keeping physically active |  | Carrying out activities to keep physically active | - 1. Goal setting behaviour   2. Problem solving   3.1 Social support unspecified  4.1 Instruction on how to perform the behaviour  5.1 Information about health consequences  5.3 Information about social and environmental consequences  5.6 Information about emotional consequences  8.1 Behavioural practice/rehearsal  8.3 Habit formation  8.7 Graded tasks  9.1 Credible source   - 1. Restructuring the physical environment | Active lifestyle, Healthy lifestyle |
|  |  |  | 1. Keeping socially active |  | Carrying out activities to keep socially active | - 1. Problem solving   3.2 Social support practical  5.1 Information about health consequences   - 1. Prompts/cues | Active lifestyle, Healthy lifestyle, Social roles |
|  |  |  | 1. Making decisions |  | Making decisions | 1.2 Problem solving  3.1 Social support unspecified  3.2 Social support practical  5.3 Information about social and environmental consequences  6.1 Demonstration of behaviour  9.1 Pros and cons | Independence |
|  |  |  | 1. Getting your message across |  | Communicating | 1.2 Problem solving  4.1 Instruction on how to perform a behaviour  5.6 Information about emotional consequences  9.1 Credible source | Independence |
|  |  |  | 1. Receiving a diagnosis of dementia |  | Adjusting to a dementia diagnosis (reducing worries etc.) | 1.2 Problem solving  3.1 Social support unspecified  4.1 Instruction on how to perform a behaviour  5.3 Information about social and environmental consequences  5.6 Information about emotional consequences  6.1 Demonstration of behaviour  9.1 Credible source | Healthy lifestyle |
|  |  |  | 1. Keeping healthy |  | Drinking water, Eating healthily, Stopping smoking, Reducing drinking | 4.1 Instruction on how to perform a behaviour  5.1 Information about health consequences  9.1 Credible source | Healthy lifestyle |
| Assessment and Tailoring | 1. Assessment | | 1. Assess ‘decision making’, using scenarios | 1 | Decision making | N/A | Independence, Maintain cognitive activities |
|  |  |  | 1. Assess ‘getting the message across’ using scenarios |  | Communicating | N/A | Independence, Social roles, Maintain cognitive activities |
|  |  |  | 1. Assess ‘social connections’ using scenarios |  | Connecting with others socially | N/A | Social roles |
|  |  |  | 1. Assess and map social connections | 1 | Support for activities | 3.1 Social support unspecified | Social roles |
|  |  |  | 1. Assessed friends and family style | 1 | Connecting with others socially | N/A | Social roles |
|  |  |  | 1. Review satisfaction with progress | 2, 3 | N/A | N/A | All (depending on what participant chooses): Independence, Healthy lifestyle, Active lifestyle, Maintain cognitive activities, Social roles |
|  | 1. Provide tailored advice | | Choose three topics to work on:   1. Keeping mentally active 2. Keeping physically active 3. Keeping socially active 4. Making decisions 5. Getting your message across 6. Receiving a diagnosis of dementia 7. Keeping healthy | 1 | Behaviours which correspond with chosen topic | See information section (3*) | All (depending on what participant chooses): Independence, Healthy lifestyle, Active lifestyle, Maintain cognitive activities, Social roles |
| PRIDE activities | 1. Plan | | 1. Set and record a goal | 1, 2, 3 | Behaviours which correspond with chosen topic | 1.1 Goal setting behaviour+ | All (depending on what participant chooses): Independence, Healthy lifestyle, Active lifestyle, Maintain cognitive activities, Social roles |
|  |  |  | 1. Set and record an action plan | 1, 2, 3 | Behaviours which correspond with chosen topic | 1.4 Action planning |  |
|  |  |  | 1. Identify facilitators and barriers, and possible solutions and record | 1, 2,3 | Behaviours which correspond with chosen topic | 1.2 Problem solving |  |
|  | 1. Do | | Discussed how to record progress | 1, 2, 3, | Behaviours which correspond with chosen topic | 2.3 Self-monitoring of behaviour | All (depending on what participant chooses): Independence, Healthy lifestyle, Active lifestyle, Maintain cognitive activities, Social roles |
|  | 1. Review | | 1. Review progress | 2, 3 | Behaviours which correspond with chosen topic | 1.5 Review behavioural goal | All (depending on what participant chooses): Independence, Healthy lifestyle, Active lifestyle, Maintain cognitive activities, Social roles |
|  |  |  | 1. Facilitators and barriers and solutions and record | 2, 3 | Behaviours which correspond with chosen topic | 1.2 Problem solving |  |
|  |  |  | 1. Reviewed and changed plan if necessary and recorded | 2,3 | Behaviours which correspond with chosen topic | - 1. Review behavioural goal |  |
|  | 1. Feedback and support | | 1. Given positive feedback | 1, 2, 3 | Behaviours which correspond with chosen topic | 2.2 Feedback on behaviour  3.1 Social support unspecified  10.4 Social reward? | All (depending on what participant chooses): Independence, Healthy lifestyle, Active lifestyle, Maintain cognitive activities, Social roles |
|  |  |  | 1. Given opportunity to ask questions |  |  |  |  |
|  |  |  | 1. Given contact details |  |  |  |  |
| *Note:*  *Key components will be referred to as appointment activities in checklists  ** These were delivered according to participants’ choice of topics | | | | | | | |

Appendix 2 – PRIDE coding guidelines for researchers

**PRIDE Coding guidelines**

**About the checklists**

There are three checklists, one for each of the PRIDE intervention sessions:

- Session 1
- Session 2
- Session 3

These checklists detail the intervention activities that should have been delivered in each session. Some activities happen in every session, others are unique to one session only (these are clearly marked in the guideline).

For session 1 and 2 there is also an additional grid with tailored activities specific to topics. Participants were asked to choose one or more of the below topics to work on:

1. Keeping mentally active
2. Keeping physically active
3. Keeping socially active
4. Making decisions
5. Getting your message across
6. Receiving a diagnosis
7. Keeping healthy

For each of these topics, certain activities are recommended in the manual (the boxes with no shading on the checklists).

**How do I fill out the checklists?**

Please:

- Read the transcript once all the way through to familiarise yourself with it
- Read the coding guidelines all the way through to familiarise yourself with them
- Read the transcript again
- Record the set number provided on the transcript in the first row of the checklist.
- Record your initials next to coder ID and put the date that you are completing the checklist.
- Go through the checklist appointment activities one by one.
- Make a note on the transcript to demonstrate evidence for each appointment activity (this will be helpful when deciding to what extent it has been delivered and when discussing your decision with the other coder). Please do this using the comment function in word.
- Please add page numbers to the checklist for each appointment activity (this will help when discussing discrepancies with the other coder)
- For every item on the checklist, please tick whether it was:
  - Done
  - Done to some extent
  - Not done
- If there is an apparent reason (e.g. dementia advice worker mentions that they have ran out of time) why this may not have been delivered or delivered to some extent, please make a note in the notes column.
- For the activity: ‘chosen topic: discussed in relation to the participant’, please turn the page and tick the activities that were carried out **for the chosen topic only**.
- Note: Only tick the boxes if activities have been done. Blank box indicates not done response and shaded box with no tick indicates N/A response.

*E.g. If the participant chose to work on ‘Keeping mentally active: look for activities that should have been delivered for that topic (it is indicated by the boxes with no shading for the topic row in both grids). Make a note on the transcript for evidence of the provider delivering those activities and then select the activities that were delivered.*

For example, if ‘Keeping mentally active’ was chosen, you would look for evidence of:

- - *‘Provided information on the benefits associated with it’*
  - *‘Provided instructions on how to do it’*
  - *‘Provided example activities for the topic’*
  - *‘Provided examples of how others do it’*
  - *‘Provided tips to overcome challenges’*

And select the activities that were delivered.

If an activity was not relevant for the chosen topic, please select not applicable.

**Note:** Shading means that this component is not in the manual, however you should still look for evidence of these components when coding in case they have been delivered

**Please code all evidence of tailored topic activities despite whether the topic is chosen (sometimes components are covered when DAWs are introducing the topics)**

**Evidence can be for more than one tailored topic at a time.**

**How do I decide which score to give?**

- Please choose **‘done’** if there is evidence in the transcript that all aspects of the activity have been delivered
- Please choose **‘done to some extent’,** if there is evidence that the activity has only been partially delivered or if the dementia advice worker could have done more.
- Please choose **‘not done’** if there is no evidence in the transcript that this activity has been delivered.

**Please see the table below for specific examples, definitions and rationale for scores per appointment activity.**

**Please note, some processes are repeated across sessions (plan, do and review), therefore it may only be suitable for the DAW to explain the process the first time. If this is the case, please code not done and write (N/A)**

**Acronyms**

DAW = Dementia advice worker

PRIDE = Promoting Independence in Dementia

| **Session 1** | | | | |
| --- | --- | --- | --- | --- |
| **Framework Component** | **Appointment Activity** | **Definition** | **Scores** | **Rationale for scores** |
| **Initial PRIDE activities** | 1. Explained what the PRIDE programme is and what it will involve. | Explanation should include:   - What PRIDE is, including what PRIDE stands for (Promoting Independence in Dementia) - What PRIDE will involve, including:   - who it will involve (DAW) and supporter)   - what the programme will involve (three sessions with DAW)   - The purpose of the programme,   - The manual | **Done** | DAW explained what the PRIDE programme is and what it will involve |
|  |  |  | **Done to some extent** | DAW explained either what the PRIDE programme is or what it will involve  Or  The DAW explained what the PRIDE programme is and some information on what PRIDE will involve (e.g. who it will involve and the manual) |
|  |  |  | **Not done** | DAW did not explain what the PRIDE programme is and what it will involve |
|  | 1. Helped the participant to complete the PRIDE profile. | PRIDE profile includes the person’s name, age, living situation, name of supporter, name of PRIDE DAW, fitness, mobility, interests and hobbies and likes and dislikes  Note: if done before session cannot code unless they say we’ve already filled in x, y and z (specific) | **Done** | DAW helped the participant to complete all of their PRIDE profile |
|  |  |  | **Done to some extent** | DAW helped the participant to complete some of their PRIDE profile |
|  |  |  | **Not done** | DAW did not help the participant to complete their PRIDE profile |
|  | 3a. Finding a balance: Provided information on how to find a balance with activities. | Provided information on how to find a balance that is outlined in the manual, including:   - rest and relaxation (may include example activities for rest and relaxation e.g. yoga/meditation/taking a bath) - having a routine, (may include having a daily routine and to do list/planning week) - setting reminders (may include examples of ways to keep track of things – diaries/sticky notes/smart phones/calendars/asking someone, tips if forget something) | **Done** | DAW provided the information provided in the manual about how to find a balance with activities (all three: rest and relaxation, having a routine, setting reminders) |
|  |  |  | **Done to some extent** | DAW provided information on some ways to find a balance with activities (1-2 of the bullet points met e.g. rest and relaxation only) or signposted to the information but not covered it |
|  |  |  | **Not done** | DAW did not provide information on how to find a balance with activities (none of the bullet points met) |
|  | 3b. Finding a balance: Helped the participant to think about which activities they find enjoyable and important, using the ‘find a balance’ grid. | Discussed the ‘find a balance grid’ – e.g. things that are enjoyable/important or not enjoyable/important  For the find a balance grid, participants will put activities in the below grid in order of importance and enjoyment   \|  \| Less important (!) \| (!!) \| More important (!!!) \| \| --- \| --- \| --- \| --- \| \| Less enjoyable ☹ \|  \|  \|  \| \| 😐 \|  \|  \|  \| \| More enjoyable ☺ \|  \|  \|  \| | **Done** | DAW helped the participant to think about which activities they find enjoyable and important (more/less) using the ‘find a balance’ grid |
|  |  |  | **Done to some extent** | DAW helped the participant to think about which activities they find enjoyable and important but did not use the ‘find a balance grid’  Or  DAW helped the participants to think about activities that were either more/less enjoyable or more/less important |
|  |  |  | **Not done** | DAW did not help the participant to think about which activities they find enjoyable and important using the ‘find a balance grid’ |
|  | 4a. How others can help: Provided information on how other people can help. | Provided information on how other people can help, as outlined in the manual. Including:   - what a support network is (explain that a support network is a group of people who can help you when you need it) - why it is important to have a support network (explain that it is important as we get older – can help you keep independence and sense of self) - how those in the support network can help (unsure of type of support – people may take over responsibility but need to do as much as you are able – finding the right balance with support – need to negotiate this) | **Done** | DAW provided information on all three of the criteria for how other people can help, as outlined in the manual |
|  |  |  | **Done to some extent** | DAW provided some information (one to two of the criteria) on how other people can help, as outlined in the manual (e.g. what a support network is but not why it is important/how others can help) |
|  |  |  | **Not done** | DAW did not provide information on how other people can help. |
|  | 4b. How others can help: Encouraged the participant to describe their current social connections. | Asking the participant to complete the social connection exercises:   1. assessing which of the social connections are most like them (tick box exercise in manual) 2. Asking them to explore who is in their support network (network exercise in manual)   **Tick box options for 1)** are:  - My closest relationships are with family who live close by  - I have close relationships with family who live close by, friends and neighbours  - I have lots of contact with family who live far away. I have lots of friends and I am involved in the wider community  - I don’t have much contact with family. I have contact with neighbours but not a lot of involvement in things outside my home  - I don’t have any close family or friends, and I don’t know my neighbours that well. I keep myself to myself.  **Asking them to explore who is in their support network, including:** Immediate family, extended family, neighbours, lifelong friends, new friends, wider community voluntary organisations, service professionals, healthcare professionals | **Done** | DAW encouraged the participant to describe their current social connections by completing both the social connection activities |
|  |  |  | **Done to some extent** | DAW encouraged the participant to describe their current social connections by completing one of the social connection activities |
|  |  |  | **Not done** | DAW did not encourage the participant to describe their current social connections |
|  | 4c. How others can help: Provided examples of how other people help others. | Provided example case studies of how people can help. For example: the case study of ‘Jill and John’ and how other people have helped them  Note: DAW may also provide examples from their own experience (e.g. ‘Other people’s support networks help them by xxx’) | **Done** | The DAW provided examples of how other people help others, and used a case study to provide an example. |
|  |  |  | **Done to some extent** | The DAW provided examples of how other people help others, but did not provide a case study as an example  Or  The DAW signposted to the case study but did not provide examples of how other people help others. |
|  |  |  | **Not done** | DAW did not provide examples of how other people help others |
|  | 5a. Keeping going: Provided information on how to choose activities to keep going. | Provided information on how to choose activities to keep going, as outlined in the manual. This includes an explanation of:   - The plan, do and review process - Making your plan - Planning an activity   - Kind of activity?   - What you would like to do (carry on, do more, try, do less)   - How (where, when, how, who and what’ - Planning to build skills/try new ways of doing things   Note: This component focuses on **explaining** the ‘plan, do, review’ process to the participant rather than making a plan | **Done** | DAW provided information on how to choose activities to keep going, as outlined in the manual |
|  |  |  | **Done to some extent** | DAW provided some but not all information provided in the manual |
|  |  |  | **Not done** | DAW did not provide information on how to choose activities to keep going |
|  | 5b. Keeping going: Provided examples of how others keep going. | Provided examples of case studies of how other people have chosen activities to keep going using the plan, do and review steps. For example, Jill and John and their plan, do and review sheets.  Note: DAW may also provide examples from their own experience (e.g. ‘Some people may keep going by xxx’ | **Done** | DAW provided examples of how other people keep going and used a case study to provide an example |
|  |  |  | **Done to some extent** | DAW provided examples of how other people keep going but did not provide a case study as an example  Or  The DAW signposted to the case study but did not provide examples of how others keep going |
|  |  |  | **Not done** | DAW did not provide examples of how other people keep going |
|  | 6. Asked the participant to choose three topics to work on. | Asked the participant to choose three topics out of the seven topics: 1) Keeping mentally active, 2) Keeping physically active, 3) Keeping socially active, 4) Making decisions, 5) Getting your message across, 6) Receiving a diagnosis, 7) Keeping healthy  Note: Code DAW behaviour not participant response. E.g. If DAW asks participant to choose three but only one or two are chosen can code ‘done’ | **Done** | DAW asked the participant to choose three topics to work on |
|  |  |  | **Done to some extent** | DAW asked the participant to choose 1 or 2 topics to work on |
|  |  |  | **Not done** | DAW did not ask the participant to choose topics to work on |
| **Plan** | 7. Helped the participant to set an activity goal. | Helped the participant to set a goal of what they would like to work on for one of the topics *(*example from manual: The goal should specify the topic they want to work and the activity that they would like to do. For example:  Topic: Making decisions  Activity: Choosing what to have for dinner  This is a more general goal than the plan made in component 9. Setting an activity goal is more a goal area e.g. reading/crosswords | **Done** | DAW helped the participant to set an activity goal |
|  |  |  | **Done to some extent** | DAW helped the participant to choose a topic or an activity  Or  DAW helped the participant to choose an activity did not follow through to setting a goal |
|  |  |  | **Not done** | DAW did not help the participant to set an activity goal |
|  | 8a. Chosen topic: Provided relevant resources for topic chosen from PRIDE manual and own sources. | Provided relevant resources refers to signposting to the relevant materials and going through them with the participant for the topic they have chosen to work on. This may also be supplemented with additional resources where necessary *(e.g. if chosen making decisions, identified the making decisions topic in the manual and given additional resources) – only code for the specific chosen topics*  E.g. If the DAW signposts to the topic in the manual and explains relevant resources for the topic, can code done. | **Done** | DAW signposted to and went through the materials in the manual and own sources for the topic chosen |
|  |  |  | **Done to some extent** | DAW signposted but did not go through the resources from the manual and own sources for the topic chosen  Or  DAW went through information relevant to the topic but did not signpost to the relevant materials in the manual and own sources |
|  |  |  | **Not done** | DAW did not signpost to or go through the relevant resources for the topic chosen |
|  | 8b. Chosen topic: Discussed in relation to the participant *(Please turn the page and provide details).* | Discussed the information in the manual which is relevant for that topic and tailored it to the participant.  ‘Resources’ refers to information from the manual and any additional resources referred to by the DAW  *Note: It may help to first decide which tailored activities have been delivered before making a judgement on this question.* If some tailored activities have been covered, this is an indicator that this component has been done. If DAW has covered tailored activities for various topics but not the specific chosen topic can code done to some extent | **Done** | DAW covered tailored activities in relation to the participant for the chosen topic |
|  |  |  | **Done to some extent** | DAW covered tailored activities in relation to the participant for other topics but not the chosen topic |
|  |  |  | **Not done** | DAW did not cover tailored activities for other topics. |
|  | 9. Made at least one plan with the participant (including where, when and how they will do the plan and who will help). | The plan should outline how they will go about the plan, including:   - where, - when, - how - with whom,   Evidence for different aspects of the plan may be found in different parts of the transcript. All four criteria should be in relation the same plan and discussed at the time of the session (deciding at a later date is not enough) | **Done** | DAW made at least one plan with the participant, which meets all four criteria |
|  |  |  | **Done to some extent** | DAW made at least one plan with the participant, which meets 1-3 of the criteria |
|  |  |  | **Not done** | DAW did not make any plans with the participant |
|  | 10. Encouraged the participant to think about what might help and what might get in the way of doing their plan(s). | Discussed with the participant what might help them to do their activity and what might get in the way  Please note: some aspects may overlap between the plan and things that help.  Note: As long as participants have chosen an activity, barriers and facilitators can be identified. | **Done** | DAW encouraged the participant to think about what might help **and** what might get in the way of doing the plan |
|  |  |  | **Done to some extent** | DAW encouraged the participant to think about what might help **or** what might get in the way of doing the plan |
|  |  |  | **Not done** | DAW did not encourage the participant to think about what might help and what might get in the way of doing the plans |
|  | 11. Encouraged the participant to think of ways to overcome problems. | If problems are identified, encouraged the participant to identify ways of overcoming the problems and coming to an agreed solution with the participant.  If it is unclear whether a solution has been agreed, code done to some extent.  For example: It is okay if DAW suggests solution if discussed with the participant and they come to an agreed solution. If not this would be done to some extent.  Only applicable if asked about barriers. If not asked about barriers: not done,. If asked and no barriers are identified (Appt activity 10.), please choose not done and write (N/A) | **Done** | DAW encouraged the participant to think of ways to overcome problems |
|  |  |  | **Done to some extent** | DAW encouraged the participant to think of some possible ways to overcome problems but did not follow through to an agreed conclusion |
|  |  |  | **Not done** | DAW did not encourage the participant to think of ways to overcome problems |
|  | 12. Recorded plan(s) on the plan sheet. | *Note: Please look for evidence of this behaviour in transcript to code.*  It must be clear that they are talking about the plan sheet– if not clear code not done.  The plan sheet must be completed for plans selected in this session only | **Done** | DAW recorded the plan(s) on the plan sheet |
|  |  |  | **Done to some extent** | DAW recorded some but not all of the plan(s) on the plan sheet |
|  |  |  | **Not done** | DAW did not record the plan on the plan(s) sheet |
| **Do** | 13. Showed the participant how to record progress between sessions. | Showed the participant the ‘do’ calendar and explains how they can use this calendar to record their progress between sessions *(e.g. recording their activities)*  *Note: Please look for evidence of this behaviour in transcript to code.*  It must be clear that they are talking about the do calendar, e.g. by referring to do calendar, activity calendar or recording activities on days of the week – if not clear code not done | **Done** | DAW showed the participant how to record progress between sessions, using the ‘do’ calendar |
|  |  |  | **Done to some extent** | DAW showed the participants how to record progress between sessions, but suggested a different method |
|  |  |  | **Not done** | DAW did not show the participants how to record progress between sessions |
| **Support** | 14. Gave positive feedback. | Gave participants positive feedback to the participants. Positive feedback should be specific to the participants’ efforts.  For example: ‘Great’, ‘Brilliant’, ‘You have done really well’. Judge based on depth of feedback and number of times | **Done** | DAW gave positive feedback to the participant 3 or more times |
|  |  |  | **Done to some extent** | DAW gave positive feedback 1-2 times |
|  |  |  | **Not done** | DAW did not give positive feedback to the participant |
|  | 15. Gave the opportunity to ask any questions or clarify any issues. | ‘Gave the opportunity’ refers to prompting the participants to ask questions or clarify issues. This could be in relation to the plans or any other aspects of the intervention.  Multiple instances of asking participants if they have any questions/whether they’d like to clarify issues can be coded as done, one example can be coded done to some extent  Example questions: ‘Is there anything you would like me to go over again?’ ‘Do you have any questions?’ | **Done** | DAW gave the opportunity to ask questions **and** clarify issues more than once |
|  |  |  | **Done to some extent** | DAW gave the opportunity to ask questions and/**or** clarify issues once |
|  |  |  | **Not done** | DAW did not give the opportunity to ask questions or clarify issues |
|  | 16. Provided contact details and explained methods of support. | Provided contact details and explained methods of support.  Methods of support may refer to contacting the DAW/ or the DAW contacting the participant between sessions via telephone  Note: If there is an indication of them giving contact details, can code | **Done** | DAW provided contact details **and** explained methods of support |
|  |  |  | **Done to some extent** | DAW provided contact details **or** explained methods of support |
|  |  |  | **Not done** | DAW did not provide contact details or explain methods of support |
| **Next step** | 17. Set a time and date for next session. | Set a time and date for the next session.  Note: If there is an indication of setting time/date in this session can code as done, if to be arranged on the phone not done | **Done** | DAW set a time **and** date for next session |
|  |  |  | **Done to some extent** | DAW set a time **or** date for next session |
|  |  |  | **Not done** | DAW did not set a time or date for next session |

| **Session 2** | | | | |
| --- | --- | --- | --- | --- |
| **Framework Component** | **Appointment Activity** | **Definition** | **Scores** | **Rationale for scores** |
| **Review** | 1. Asked participant about his/her progress since the last session. | Asked the participant about their progress since the last session *(e.g. how it went when they tried to carry out their activities)* | **Done** | DAW asked the participant about their progress in relation to their activities since last session |
|  |  |  | **Done to some extent** | DAW asked the person about their progress but since last session but not specifically in relation to their activities |
|  |  |  | **Not done** | DAW did not ask participants about their progress since last session |
|  | 1. Discussed what helped and what got in the way of participant’s progress. | Discussed with the participant what helped them to carry out the activity and what got in the way of carrying out the activity  Barriers and facilitators can be specific or more general | **Done** | DAW discussed with the participant what helped **and** what got in the way of progress |
|  |  |  | **Done to some extent** | DAW discussed with the participant what helped **or** what got in the way of progress |
|  |  |  | **Not done** | DAW did not discuss with the participant what helped or what got in the way of progress |
|  | 1. If problems were identified, discussed ways to overcome them. | If problems are identified, encouraged the participant to identify ways of overcoming the problems and coming to an agreed solution with the participant.  If it is unclear whether a solution has been agreed, code done to some extent.  For example: It is okay if DAW suggests solution if discussed with the participant and they come to an agreed solution. If not this would be done to some extent.  Only applicable if barriers are identified (Appt activity 2). If not, please choose not done and write (N/A) | **Done** | DAW discussed with the participant ways to overcome problems |
|  |  |  | **Done to some extent** | DAW discussed with the participant some possible ways to overcome problems but did not follow through to an agreed conclusion |
|  |  |  | **Not done** | DAW did not discuss with the participant ways to overcome problems |
|  | 1. Discussed and changed plan if needed. | Discussed the plan with the participant and asked if they would like to change or carry on with it, and changed it as necessary | **Done** | DAW discussed and changed the plan, with the participant, if needed, or decided to keep the plan the same |
|  |  |  | **Done to some extent** | DAW discussed wanting to change the plan with the participant, but did not change it  Or  DAW changed the plan but did not discuss this with the participant |
|  |  |  | **Not done** | DAW did not discuss or change the plan with the participant, if needed |
|  | 1. Recorded review using the review sheet. | *Note: Please look for evidence of this behaviour in transcript to code.*  It must be clear that they are talking about the review sheet– if not clear code not done. | **Done** | DAW recorded the review on the review sheet |
|  |  |  | **Done to some extent** | DAW recorded some of the review on the review sheet |
|  |  |  | **Not done** | DAW did not record the review on the review sheet |
|  | 1. Assessed participant’s satisfaction with their plan(s). | Assessed how satisfied the participant was with their plan. The DAW should specifically ask how happy they are with their plan/progress  E.g.   - ‘How happy are you with your plan?’ - ‘How happy are you with the progress you have made?’ | **Done** | DAW assessed the participant’s satisfaction with the plan(s) |
|  |  |  | **Done to some extent** | DAW assessed satisfaction with a specific aspect of the plan, but not the plan in general |
|  |  |  | **Not done** | DAW did not assess satisfaction with the plan(s) |
| **Plan** | 1. Helped the participant to set an activity goal. | Helped the participant to set a goal of what they would like to work on for one of the topics *(*example from manual: The goal should specify the topic they want to work and the activity that they would like to do. For example:  Topic: Making decisions  Activity: Choosing what to have for dinner  This is a more general goal than the plan made in component 9. Setting an activity goal is more a goal area e.g. reading/crosswords | **Done** | DAW helped the participant to set an activity goal |
|  |  |  | **Done to some extent** | DAW helped the participant to choose a topic or an activity  Or  DAW helped the participant to choose an activity did not follow through to setting a goal |
|  |  |  | **Not done** | DAW did not help the participant to set an activity goal |
|  | 8a. Chosen topic: Provided relevant resources for topic chosen from PRIDE manual and own sources. | Provided relevant resources refers to signposting to the relevant materials and going through them with the participant for the topic they have chosen to work on. This may also be supplemented with additional resources where necessary *(e.g. if chosen making decisions, identified the making decisions topic in the manual and given additional resources) – only code for the specific chosen topics*  E.g. If the DAW signposts to the topic in the manual and explains relevant resources for the topic, can code done. | **Done** | DAW signposted to and went through the materials in the manual and own sources for the topic chosen |
|  |  |  | **Done to some extent** | DAW signposted but did not go through the resources from the manual and own sources for the topic chosen  Or  DAW went through information relevant to the topic but did not signpost to the relevant materials in the manual and own sources |
|  |  |  | **Not done** | DAW did not signpost to or go through the relevant resources for the topic chosen |
|  | 8b. Chosen topic: Discussed in relation to the participant *(Please turn the page and provide details).* | Discussed the information in the manual which is relevant for that topic and tailored it to the participant.  ‘Resources’ refers to information from the manual and any additional resources referred to by the DAW  *Note: It may help to first decide which tailored activities have been delivered before making a judgement on this question.* If some tailored activities have been covered, this is an indicator that this component has been done. If DAW has covered tailored activities for various topics but not the specific chosen topic can code done to some extent | **Done** | DAW covered tailored activities in relation to the participant for the chosen topic |
|  |  |  | **Done to some extent** | DAW covered tailored activities in relation to the participant for other topics but not the chosen topic |
|  |  |  | **Not done** | DAW did not cover tailored activities for other topics. |
|  | 1. Made at least one plan with the participant (including where, when and how they will do the plan and who will help). | The plan should outline how they will go about the plan, including:   - where, - when, - how - with whom,   Evidence for different aspects of the plan may be found in different parts of the transcript. All four criteria should be in relation the same plan and discussed at the time of the session (deciding at a later date is not enough) | **Done** | DAW made at least one plan with the participant, which meets all four criteria |
|  |  |  | **Done to some extent** | DAW made at least one plan with the participant, which meets 1-3 of the criteria |
|  |  |  | **Not done** | DAW did not make any plan with the participant |
|  | 1. Encouraged the participant to think about what might help and what might get in the way of doing their plan(s). | Discussed with the participant what might help them to do their activity and what might get in the way  Please note: some aspects may overlap between the plan and things that help.  Note: As long as participants have chosen an activity, barriers and facilitators can be identified. | **Done** | DAW encouraged the participant to think about what might help **and** what might get in the way of doing the plan |
|  |  |  | **Done to some extent** | DAW encouraged the participant to think about what might help **or** what might get in the way of doing the plan |
|  |  |  | **Not done** | DAW did not encourage the participant to think about what might help and what might get in the way of doing the plans |
|  | 1. Encouraged the participant to think of ways to overcome problems. | If problems are identified, encouraged the participant to identify ways of overcoming the problems and coming to an agreed solution with the participant.  If it is unclear whether a solution has been agreed, code done to some extent.  For example: It is okay if DAW suggests solution if discussed with the participant and they come to an agreed solution. If not this would be done to some extent.  Only applicable if asked about barriers. If not asked about barriers: not done. If asked and no barriers are identified (Appt activity 10.), please choose not done and write (N/A) | **Done** | DAW encouraged the participant to think of ways to overcome problems |
|  |  |  | **Done to some extent** | DAW encouraged the participant to think of some possible ways to overcome problems but did not follow through to an agreed conclusion |
|  |  |  | **Not done** | DAW did not encourage the participant to think of ways to overcome problems |
|  | 1. Recorded plan(s) on the plan sheet. | *Note: Please look for evidence of this behaviour in transcript to code.*  It must be clear that they are talking about the plan sheet– if not clear code not done.  The plan sheet must be completed for plans selected in this session only | **Done** | DAW recorded the plan(s) on the plan sheet |
|  |  |  | **Done to some extent** | DAW recorded some but not all of the plan(s) on the plan sheet |
|  |  |  | **Not done** | DAW did not record the plan on the plan(s) sheet |
| **Do** | 1. Showed the participant how to record progress between sessions. | Showed the participant the ‘do’ calendar and explains how they can use this calendar to record their progress between sessions *(e.g. recording their activities)*  *Note: Please look for evidence of this behaviour in transcript to code.*  It must be clear that they are talking about the do calendar, e.g. by referring to do calendar, activity calendar or recording activities on days of the week – if not clear code not done | **Done** | DAW showed the participant how to record progress between sessions, using the ‘do’ calendar |
|  |  |  | **Done to some extent** | DAW showed the participants how to record progress between sessions, but suggested a different method |
|  |  |  | **Not done** | DAW did not show the participants how to record progress between sessions |
| **Support** | 1. Gave positive feedback. | Gave participants positive feedback to the participants. Positive feedback should be specific to the participants’ efforts.  For example: ‘Great’, ‘Brilliant’, ‘You have done really well’. Judge based on depth of feedback and number of times | **Done** | DAW gave positive feedback to the participant 3 or more times |
|  |  |  | **Done to some extent** | DAW gave positive feedback 1-2 times |
|  |  |  | **Not done** | DAW did not give positive feedback to the participant |
|  | 1. Gave the opportunity to ask any questions or clarify any issues. | ‘Gave the opportunity’ refers to prompting the participants to ask questions or clarify issues. This could be in relation to the plans or any other aspects of the intervention.  Multiple instances of asking participants if they have any questions/whether they’d like to clarify issues can be coded as done, one example can be coded done to some extent  Example questions: ‘Is there anything you would like me to go over again?’ ‘Do you have any questions?’ | **Done** | DAW gave the opportunity to ask questions **and** clarify issues more than once |
|  |  |  | **Done to some extent** | DAW gave the opportunity to ask questions and/**or** clarify issues once |
|  |  |  | **Not done** | DAW did not give the opportunity to ask questions or clarify issues |
|  | 1. Provided contact details and explained methods of support. | Provided contact details and explained methods of support.  Note: If there is an indication of them giving contact details, can code | **Done** | DAW provided contact details **and** explained methods of support |
|  |  |  | **Done to some extent** | DAW provided contact details **or** explained methods of support |
|  |  |  | **Not done** | DAW did not provide contact details or explain methods of support |
| **Next steps** | 1. Set a time and date for next session. | Set a time and date for the next session.  Note: If there is an indication of setting time/date in this session can code as done, if to be arranged on the phone not done | **Done** | DAW set a time **and** date for next session |
|  |  |  | **Done to some extent** | DAW set a time **or** date for next session |
|  |  |  | **Not done** | DAW did not set a time or date for next session |

| **Session 3** | | | | |
| --- | --- | --- | --- | --- |
| **Framework Component** | **Appointment Activity** | **Definition** | **Scores** | **Rationale for scores** |
| **Review** | 1. Asked the participant about his/her progress since the last session. | Asked the participant about their progress since the last session *(e.g. how it went when they tried to carry out their activities)* | **Done** | DAW asked the participant about their progress in relation to their activities since last session |
|  |  |  | **Done to some extent** | DAW asked the person about their progress but since last session but not specifically in relation to their activities |
|  |  |  | **Not done** | DAW did not ask participants about their progress since last session |
|  | 1. Discussed what helped and what got in the way of the participant’s progress. | Discussed with the participant what helped them to carry out the activity and what got in the way of carrying out the activity  Barriers and facilitators can be specific or more general | **Done** | DAW discussed with the participant what helped **and** what got in the way of progress |
|  |  |  | **Done to some extent** | DAW discussed with the participant what helped **or** what got in the way of progress |
|  |  |  | **Not done** | DAW did not discuss with the participant what helped or what got in the way of progress |
|  | 1. If problems were identified, discussed ways to overcome them. | If problems are identified, encouraged the participant to identify ways of overcoming the problems and coming to an agreed solution with the participant.  If it is unclear whether a solution has been agreed, code done to some extent.  For example: It is okay if DAW suggests solution if discussed with the participant and they come to an agreed solution. If not this would be done to some extent.  Only applicable if barriers are identified (Appt activity 2). If not, please choose not done and write (N/A) | **Done** | DAW discussed with the participant ways to overcome problems |
|  |  |  | **Done to some extent** | DAW discussed with the participant some possible ways to overcome problems but did not follow through to an agreed conclusion |
|  |  |  | **Not done** | DAW did not discuss with the participant ways to overcome problems |
|  | 1. Discussed and changed plan(s) if needed. | Discussed the plan with the participant and asked if they would like to change or carry on with it, and changed it as necessary | **Done** | DAW discussed and changed the plan, with the participant, if needed, or decided to keep the plan the same |
|  |  |  | **Done to some extent** | DAW discussed wanting to change the plan with the participant, but did not change it  Or  DAW changed the plan but did not discuss this with the participant |
|  |  |  | **Not done** | DAW did not discuss or change the plan with the participant, if needed |
|  | 1. Recorded review using the review sheet. | *Note: Please look for evidence of this behaviour in transcript to code.*  It must be clear that they are talking about the review sheet– if not clear code not done. | **Done** | DAW recorded the review on the review sheet |
|  |  |  | **Done to some extent** | DAW recorded some of the review on the review sheet |
|  |  |  | **Not done** | DAW did not record the review on the review sheet |
|  | 1. Assessed participant’s satisfaction with their plan(s). | Assessed how satisfied the participant was with their plan. The DAW should specifically ask how happy they are with their plan/progress. E.g.   - ‘How happy are you with your plan?’ - ‘How happy are you with the progress you have made?’ | **Done** | DAW assessed the participant’s satisfaction with the plan(s) |
|  |  |  | **Done to some extent** | DAW assessed satisfaction with a specific aspect of the plan, but not the plan in general |
|  |  |  | **Not done** | DAW did not assess satisfaction with the plan(s) |
| **Plan: going forward** | 1. Helped the participant to set an activity goal to work on after the programme. | Helped the participant to set a new goal which specifies what they want to work on in the long term after the programme.  This plan may be more general than plans made in previous sessions | **Done** | DAW helped the participant to set an activity goal to work on after the programme |
|  |  |  | **Done to some extent** | DAW helped the participants to think about an activity they might like to work on after the programme, but did not follow through to an agreed conclusion |
|  |  |  | **Not done** | DAW did not help the participant to set an activity goal to work on after the programme |
|  | 1. Encouraged the participant to think about what might help and what might get in the way of doing their plan(s). | Encouraged the participant to think about things that might help and get in the way of these long-term plans.  Barriers and facilitators can be specific or more general | **Done** | DAW encouraged the participant to think about what might help **and** what might get in the way of doing the plan |
|  |  |  | **Done to some extent** | DAW encouraged the participant to think about what might help **or** what might get in the way of doing the plan |
|  |  |  | **Not done** | DAW did not encourage the participant to think about what might help and what might get in the way of doing the plans |
|  | 1. Encouraged the participant to think of ways to overcome problems. | If problems are identified, encouraged the participant to identify ways of overcoming the problems and coming to an agreed solution with the participant.  If it is unclear whether a solution has been agreed, code done to some extent.  For example: It is okay if DAW suggests solution if discussed with the participant and they come to an agreed solution. If not this would be done to some extent.  Only applicable if asked about barriers. If not asked about barriers: not done. If asked and no barriers are identified (Appt activity 10.), please choose not done and write (N/A) | **Done** | DAW encouraged the participant to think of ways to overcome problems |
|  |  |  | **Done to some extent** | DAW encouraged the participant to think of some possible ways to overcome problems but did not follow through to an agreed conclusion |
|  |  |  | **Not done** | DAW did not encourage the participant to think of ways to overcome problems |
|  | 1. Recorded plan(s) going forward on the ‘things to take forward’ sheet. | *Note: Please look for evidence of this behaviour in transcript to code.*  It must be clear that they are talking about the things to take forward sheet– if not clear code not done. | **Done** | DAW recorded the plan(s) on the ‘things to take forward’ sheet |
|  |  |  | **Done to some extent** | DAW recorded some but not all of the plan(s) on the ‘things to take forward’ sheet |
|  |  |  | **Not done** | DAW did not record the plan on the ‘things to take forward’ sheet |
| **Support** | 1. Gave positive feedback. | Gave participants positive feedback to the participants. Positive feedback should be specific to the participants’ efforts.  For example: ‘Great’, ‘Brilliant’, ‘You have done really well’. Judge based on depth of feedback and number of times | **Done** | DAW gave positive feedback to the participant 3 or more times |
|  |  |  | **Done to some extent** | DAW gave positive feedback 1-2 times |
|  |  |  | **Not done** | DAW did not give positive feedback to the participant |
|  | 1. Gave the opportunity to ask any questions or clarify any issues. | ‘Gave the opportunity’ refers to prompting the participants to ask questions or clarify issues. This could be in relation to the plans or any other aspects of the intervention.  Multiple instances of asking participants if they have any questions/whether they’d like to clarify issues can be coded as done, one example can be coded done to some extent  Example questions: ‘Is there anything you would like me to go over again?’ ‘Do you have any questions?’ | **Done** | DAW gave the opportunity to ask questions **and** clarify issues more than once |
|  |  |  | **Done to some extent** | DAW gave the opportunity to ask questions and/**or** clarify issues once |
|  |  |  | **Not done** | DAW did not give the opportunity to ask questions or clarify issues |

| **Session 1 & 2: Tailored grid** | | | | |
| --- | --- | --- | --- | --- |
| **Framework** | **Appointment activity** | **Definition** | **Scores** | **Rationale for scores** |
| Tailored grid: | Provided information on the benefits associated with it  *Note: ‘It’ refers to the chosen topic/activity* | Provides information on the benefits of working on the chosen topic/activity. Specific benefits should be given for each topic. Benefits can include: cognitive functioning, physical, social and mental wellbeing.  **Relevant for all seven topics**. Some benefits listed in the manual for each topic that may be covered.   1. **Keeping mentally active:** Cognitive functioning 2. **Keeping physically active:** Physical, mental and social health. 3. **Keeping socially active:** Physical and mental health and cognitive functioning 4. **Making decisions:** independence 5. **Getting your message across:** Well-being 6. **Receiving a diagnosis:** Clarity on next steps/planning/support 7. **Keeping healthy:** Physical health and cognitive functioning   Other benefits may also be discussed and should be taken into consideration when coding.  If greyed out but evident in transcript, still tick | Done | Provided information on the benefits associated with the participant’s choice of topic. |
|  |  |  | Not done | Did not provide Information on the benefits associated with the participant’s choice of topic |
|  |  |  | Not applicable | This activity was not relevant for the topic chosen. |
|  | Provided information on how dementia can affect it  *Note: ‘It’ refers to the chosen topic/activity* | Provided information on how dementia can affect the chosen topic/activity. Specific examples for how dementia can affect it must be given, e.g. ‘([topic] can be affected by dementia in many ways) is not sufficient.  Relevant for: 4) Making decisions 5) Getting your message across  Some information that may be covered for each topic, from the manual:  **4) Making decisions:** Information about dementia not stopping the person from having a say, types of decisions that the person makes changing, and information about decisions that the person may never have been involved in  **5) Getting your message across:** Information about the person expressing themselves and understanding information, e.g. trouble thinking of words/objects/people, repetition, difficulty tracking conversations.  If greyed out but evident in transcript, still tick | Done | Provided information on how dementia can affect the topic chosen by the participant |
|  |  |  | Not done | Did not provide Information on how dementia can affect the topic chosen by the participant |
|  |  |  | Not applicable | This activity was not relevant for the topic chosen. |
|  | Assessed participant’s current style (decision making/communication) | Assessed the participant’s current decision making or communication style.  Relevant for: 4) Making decisions, 5) Getting your message across  In both of these topics, the dementia advice worker will assess the person’s style using the flow diagrams in the manual.  If greyed out but evident in transcript, still tick | Done | Assessed the participant’s current style |
|  |  |  | Not done | Did not assess the participant’s current style |
|  |  |  | Not applicable | This activity was not relevant for the topic chosen. |
|  | Identified potential challenges | Identified potential challenges for working on that topic/chosen activity. Specific challenges must be identified e.g. (there are many challenges associated with [topic]) is not sufficient.  Relevant for: 4) Making decisions, 5) Getting your message across, 6) Receiving a diagnosis  Some challenges that may be identified for each topic:  **4) Making decisions:** May include risky activities, feeling left out of decisions, being limited by other people’s decisions, lack of confidence making decisions and trouble making decisions  **5) Getting your message across:** May include keeping involved, confidence, talking to people about feelings, talking to people about having dementia and health issues  **6) Receiving a diagnosis:** May include worries about receiving a diagnosis and sharing a diagnosis  If greyed out but evident in transcript, still tick | Done | Identified potential challenges for the participant’s chosen topic |
|  |  |  | Not done | Did not identify potential challenges for the participant’s chosen topic |
|  |  |  | Not applicable | This activity was not relevant for the topic chosen. |
|  | Provided information on resources | Provided information and resources for the participant to find further information  Relevant for: 7) Keeping healthy  Relevant resources may include: general health resources, heart health resources, diabetes resources, lifestyle resources (eating and drinking, managing weight, sleep, managing worries, taking care of teeth), smoking and drinking  If greyed out but evident in transcript, still tick | Done | Provided information on resources for the participant’s chosen topic |
|  |  |  | Not done | Did not provide information on resources for the participant’s chosen topic |
|  |  |  | Not applicable | This activity was not relevant for the topic chosen. |
|  | Provided instructions on how to do it  *Note: ‘It’ refers to the chosen topic/activity* | Provided instructions on how the participant could achieve their chosen activity/make progress with that topic  Relevant for: 1) Keeping mentally active, 2) Keeping physically active, 3) Keeping socially active  For these topics, instructions which explain how the person can participate in mental, physical or social activities should be provided. This could include the difficulty of the activity, equipment and safety  If greyed out but evident in transcript, still tick | Done | Provided instructions on how to do the chosen topic |
|  |  |  | Not done | Did not provide instructions on how to do the chosen topic |
|  |  |  | Not applicable | This activity was not relevant for the topic chosen. |
|  | Provided example activities for this topic | Provided examples of activities for the chosen topic.  Relevant for: 1) Keeping mentally active, 2) Keeping physically active, 3) Keeping socially active  For these topics, example activities may be provided, for example: puzzles/reading/computer use/CST (mentally active), walking, swimming, chair exercises (physically activity), and volunteering, singing groups, courses, cinema (Socially active)  If greyed out but evident in transcript, still tick | Done | Provided example activities for the chosen topic |
|  |  |  | Not done | Did not provide example activities for the chosen topic |
|  |  |  | Not applicable | This activity was not relevant for the topic chosen. |
|  | Provided examples of how others do it  *Note: ‘It’ refers to the chosen topic/activity* | Provided examples or case studies of how others do the chosen activity  Relevant for: 1) Keeping mentally active, 2) Keeping physically active, 3) Keeping socially active, 4) Making decisions, 5) Getting your message across and 6) Receiving a diagnosis  **1)Keeping mentally active:** Explored case studies for how others keep mentally active, for example (Simon, Alan and Anna)  **2)Keeping physically active:** Explored case studies for how others keep physically active, for example: Kate and James, Aki and Haya, Lana  **3) Keeping socially active:** Explored case studies for how others keep socially active, for example: Andrew, Mary, Rachel.  **4) Making decisions:** Explored the case studies provided in the manual for the relevant challenge to see how others make decisions (e.g. David & Elsie, Sandra, Curtis, & Roberts’ stories  **5) Getting the message across:** Explored the case studies provided in the manual for examples of how others get the message across (e.g. Violet, Gladys, Harry, Irene, Phillip and June)  **6) Receiving a diagnosis:** Explored the case studies provided in the manual for examples of how others received their diagnosis (e.g. Terry, Josephine, Claudia)  If greyed out but evident in transcript, still tick | Done | Provided examples of how others do the chosen topic |
|  |  |  | Not done | Did not provide examples of how others do the chosen topic |
|  |  |  | Not applicable | This activity was not relevant for the topic chosen. |
|  | Provided tips on how others provide support | Provided tips (guidance) on how other people can support the participant to achieve their chosen activity/topic. Note: It must be clear which topic(s) they are talking about  Relevant for: 1) Keeping mentally active, 2) Keeping physically active, 3) Keeping socially active, 4) Making decisions, 5) Getting your message across, 6) Receiving a diagnosis  Providing tips on how others can help you with these activities, for example asking family members/friends to support or join in with an activity  **1, 2 and 3) Keeping mentally active, physically active and socially active:** Provided tips to explain how others can support in this, for example asking family members or friends to support/ go with them to an activity  **4) Making decisions: Tips** on how others can provide a little support, backup, enable rather than limit, offer choices, help with part of a task, consider options together and know which decisions are important  **5) Getting your message across:** Tips on how you can use support from healthcare professionals, family and friends to get your message across.  **6) Receiving a diagnosis:** Tips on how support groups and healthcare professionals, family and friends can support your with receiving a diagnosis.  If greyed out but evident in transcript, still tick | Done | Provided tips on how others can provide support for the chosen topic |
|  |  |  | Not done | Did not provide tips on how others can provide support for the chosen topic |
|  |  |  | Not applicable | This activity was not relevant for the topic chosen. |
|  | Provided examples of how others overcome challenges | Provided examples or case studies of how other people have overcome difficulties to achieve the chosen activity/topic  Relevant for: 4) Making decisions, 5) Getting your message across, 6) Receiving a diagnosis  **4) Making decisions:** Explored the case studies provided in the manual for the relevant challenge to see how others have overcome challenges (e.g. David & Elsie, Sandra, Curtis, & Roberts’ stories  **5) Getting the message across:** Explored the case studies provided in the manual for relevant challenges to see how overs may have overcome challenge (e.g. Ian)  **6) Receiving a diagnosis:** Explored the case studies provided in the manual to see how others have overcome these challenges (e.g. Rosa, Ben & Sade, Zach, Ali, Jay and Gita)  If greyed out but evident in transcript, still tick | Done | Provided examples of how others overcome challenges relating to the chosen topic |
|  |  |  | Not done | Did not provide examples of how others overcome challenges relating to the chosen topic |
|  |  |  | Not applicable | This activity was not relevant for the topic chosen. |
|  | Provided tips to overcome challenges | Provided tips (guidance) to the participant on how they can overcome problems.  Relevant for: 1) Keeping mentally active, 2) Keeping physically active, 3) Keeping socially active, 4) Making decisions, 5) Getting your message across, 6) Receiving a diagnosis   1. **Keeping mentally active:** Covered tips of how to overcome challenges for keeping mentally active, including doing activities at home instead of outside, using technological aids, making notes, 2. **Keeping physically active:** Covered tips of how to overcome challenges for keeping physically active, for example: setting goals, using technology and other equipment   **3) Keeping socially active:** Covered tips of how to overcome challenges, for example: prompts, making list, using equipment, e.g. timers.  **4) Making decisions:** Covered tips of how to identify the challenges related to decision making e.g. changing routine, talking to other people to negotiate roles/activities,  **5) Getting your message across:** Covered tips which may include talking to people to show you want to be in the conversation, looking at communication resources, talking to a health professional, having patience, using reminders,  **6) Receiving a diagnosis:** Covered tips including getting support, finding out about dementia, speaking openly to others, raising awareness, humour, looking at situation from all points of view)  If greyed out but evident in transcript, still tick | Done | Provided tips to overcome challenges relating to the chosen topic |
|  |  |  | Not done | Did not provide tips to overcome challenges relating to the chosen topic |
|  |  |  | Not applicable | This activity was not relevant for the topic chosen. |


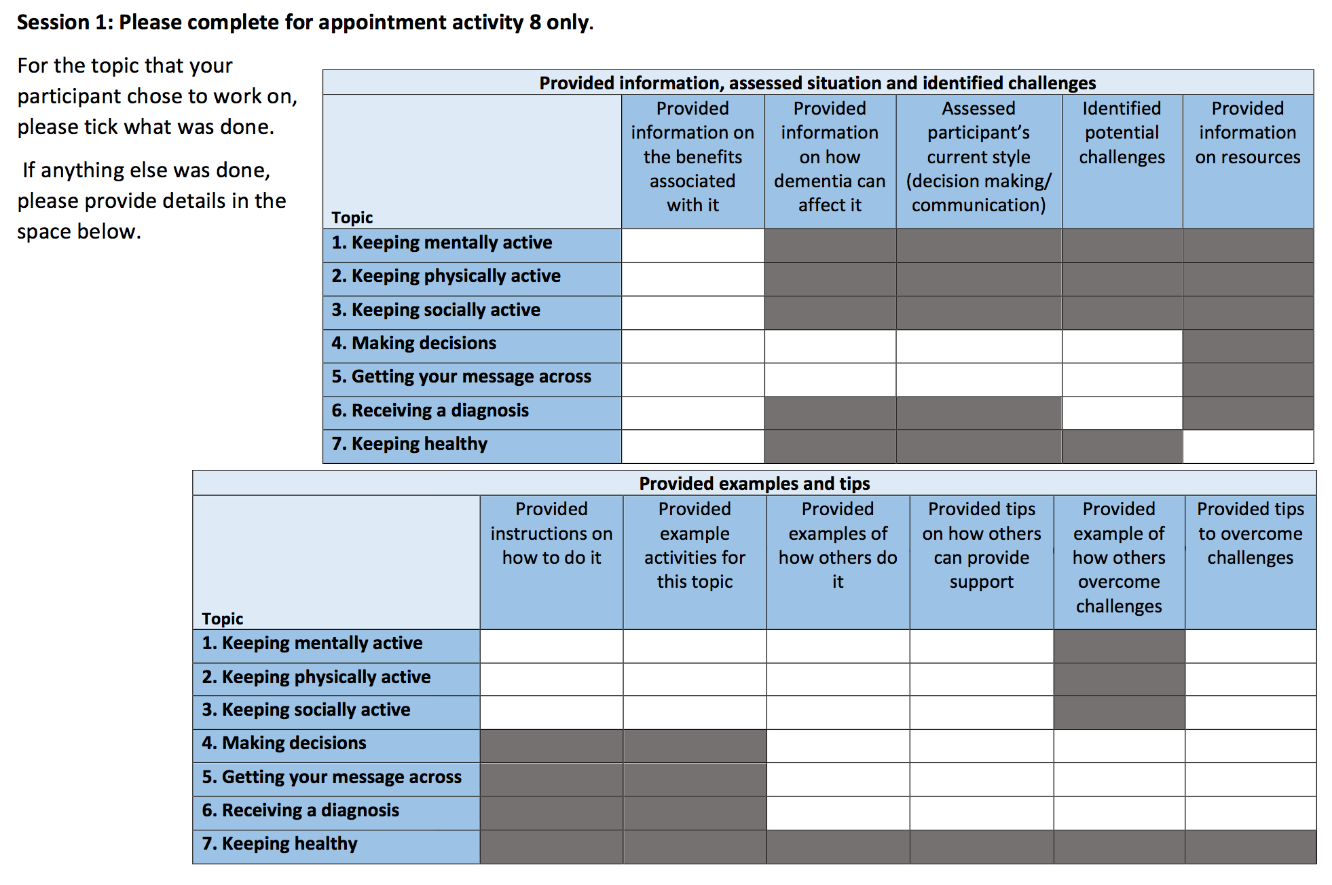

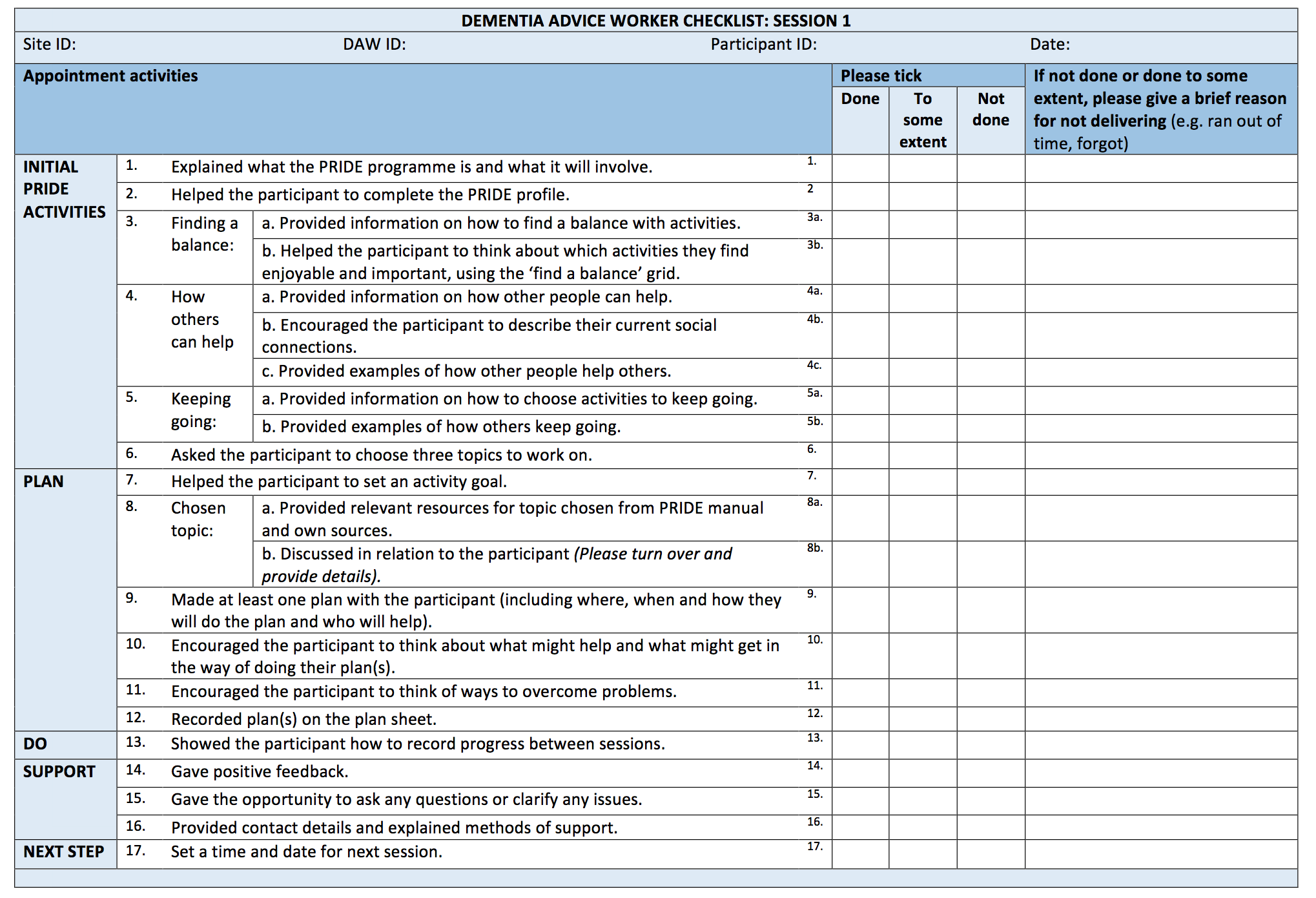
Appendix 3 – Provider/researcher fidelity checklists, Sessions 1-3


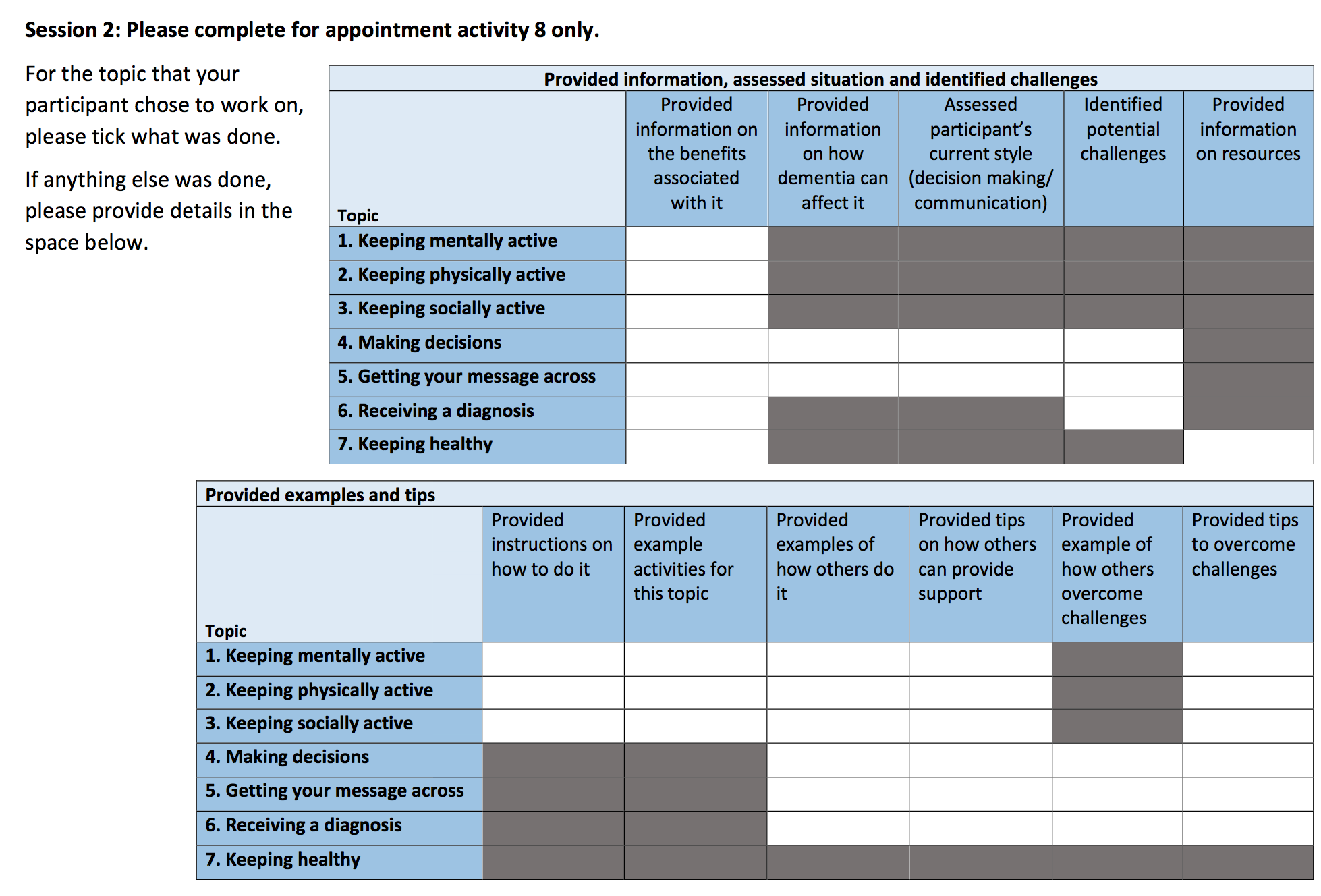

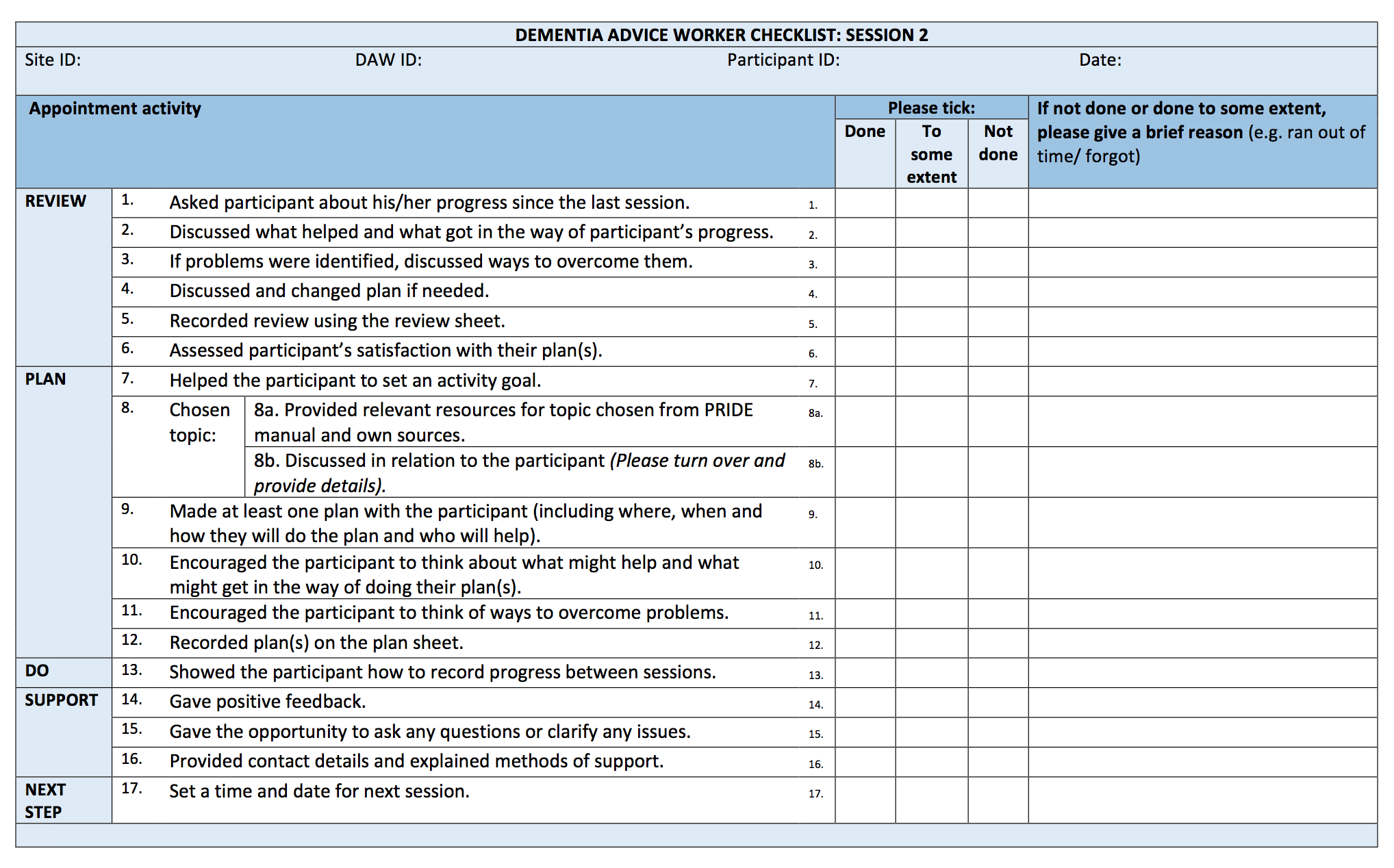


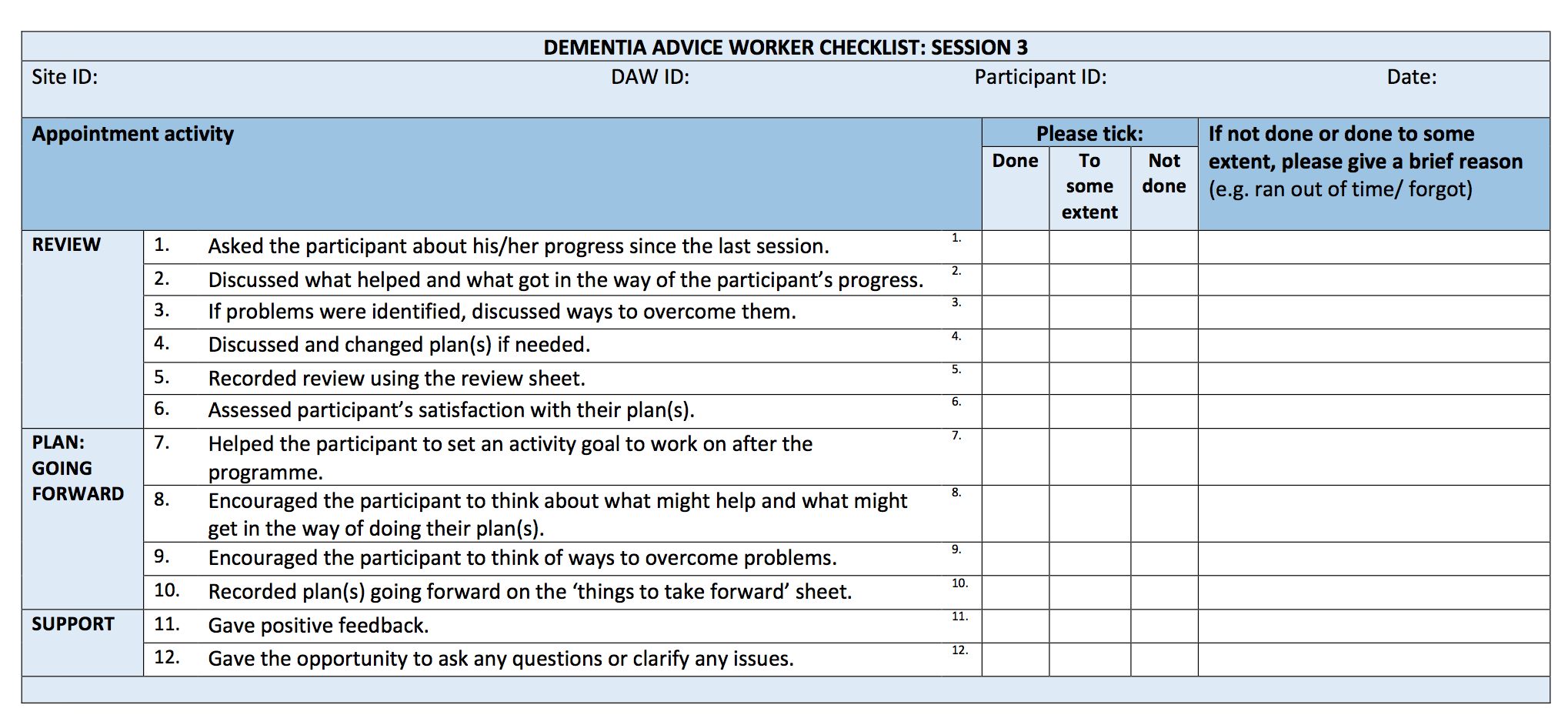


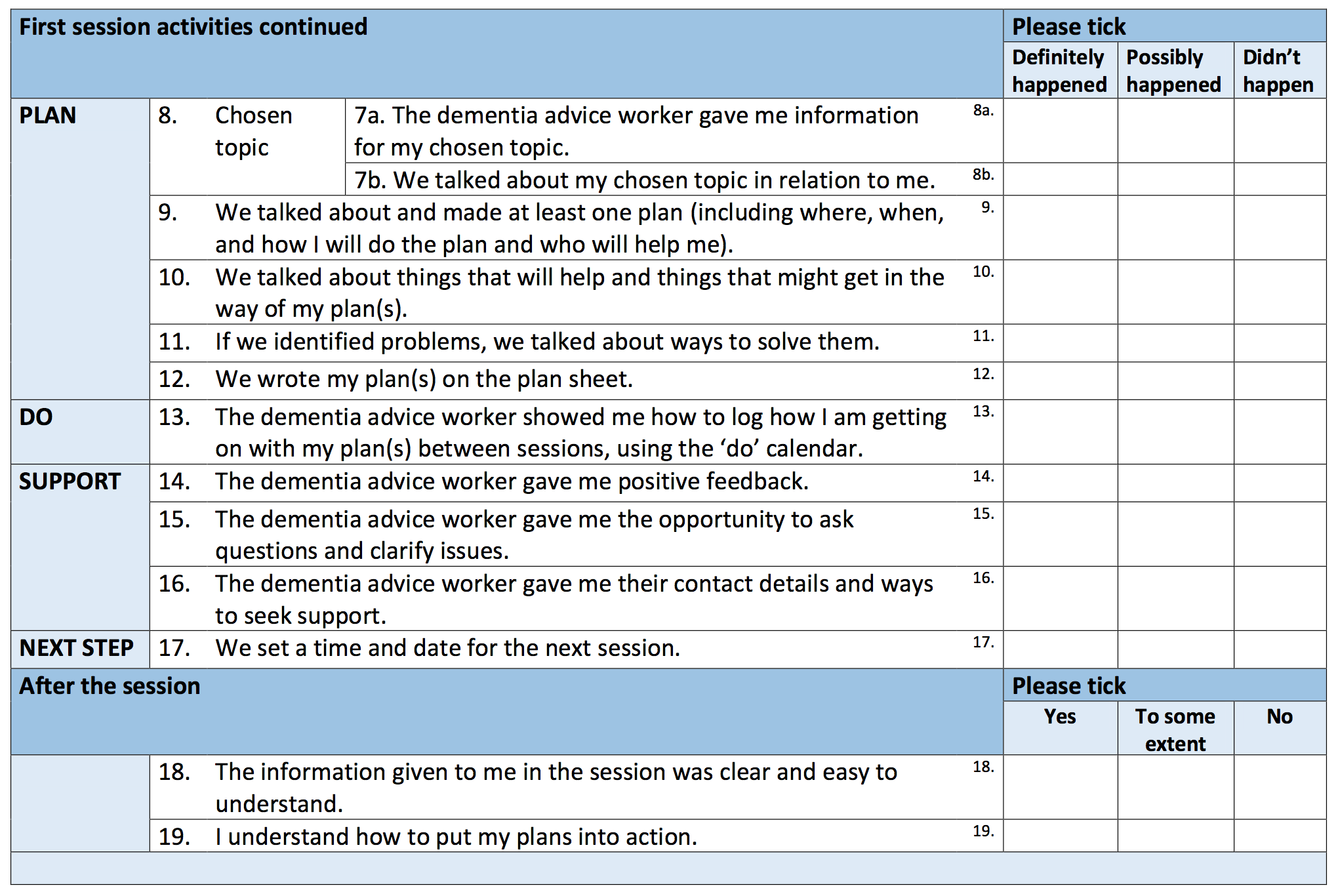

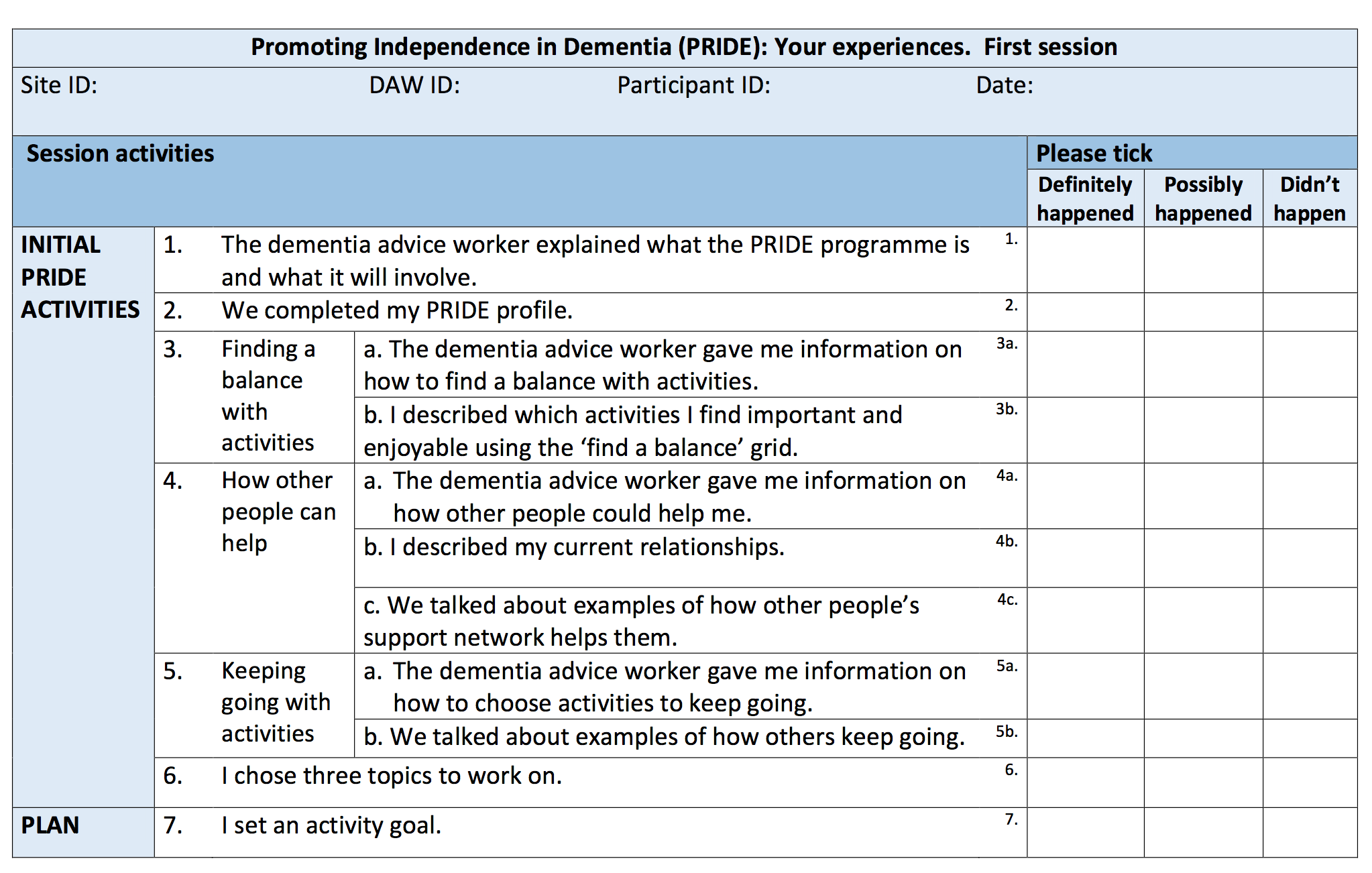
Appendix 4 – Participant ‘your experience’ fidelity checklists, Sessions 1-3


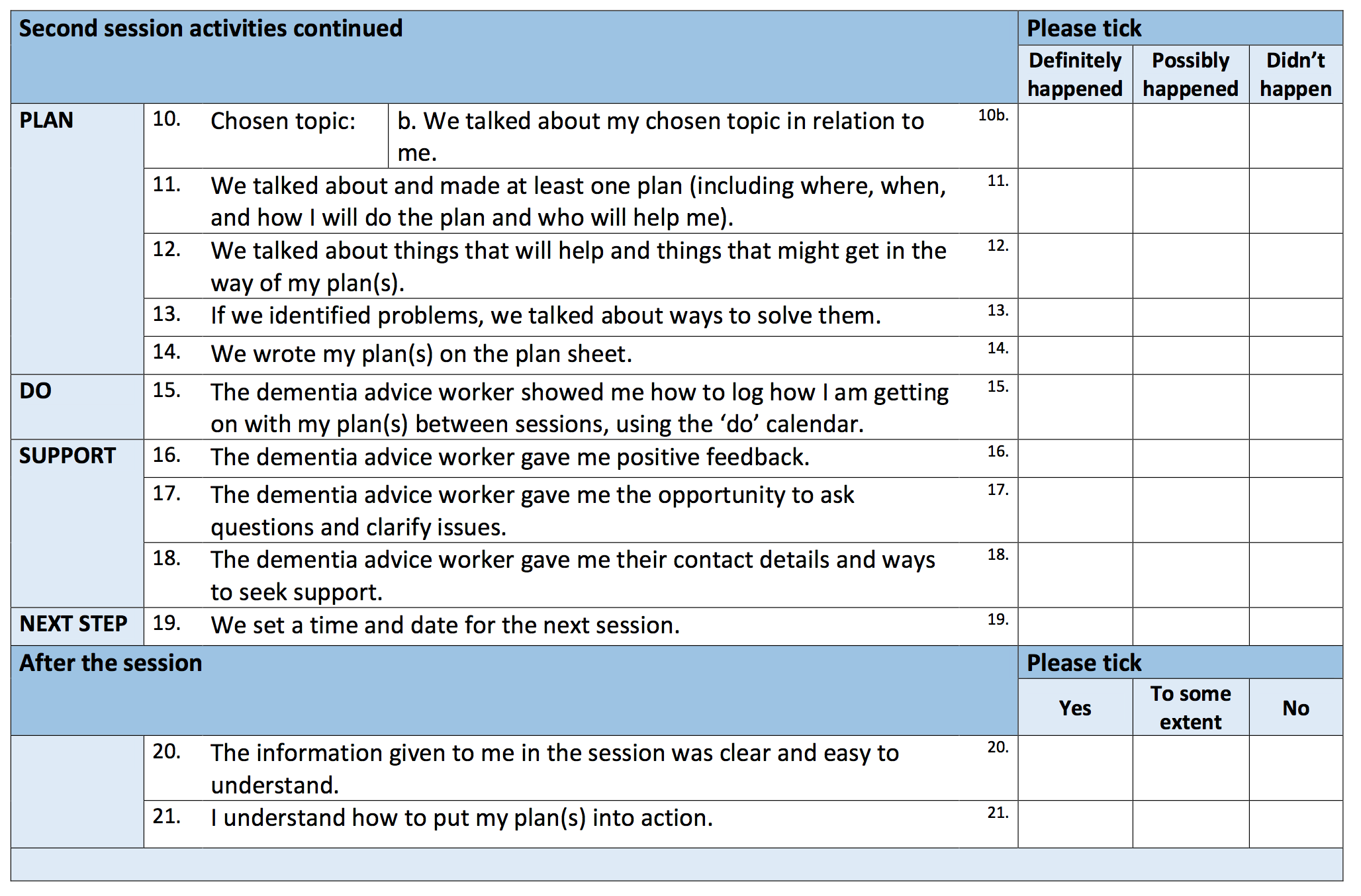

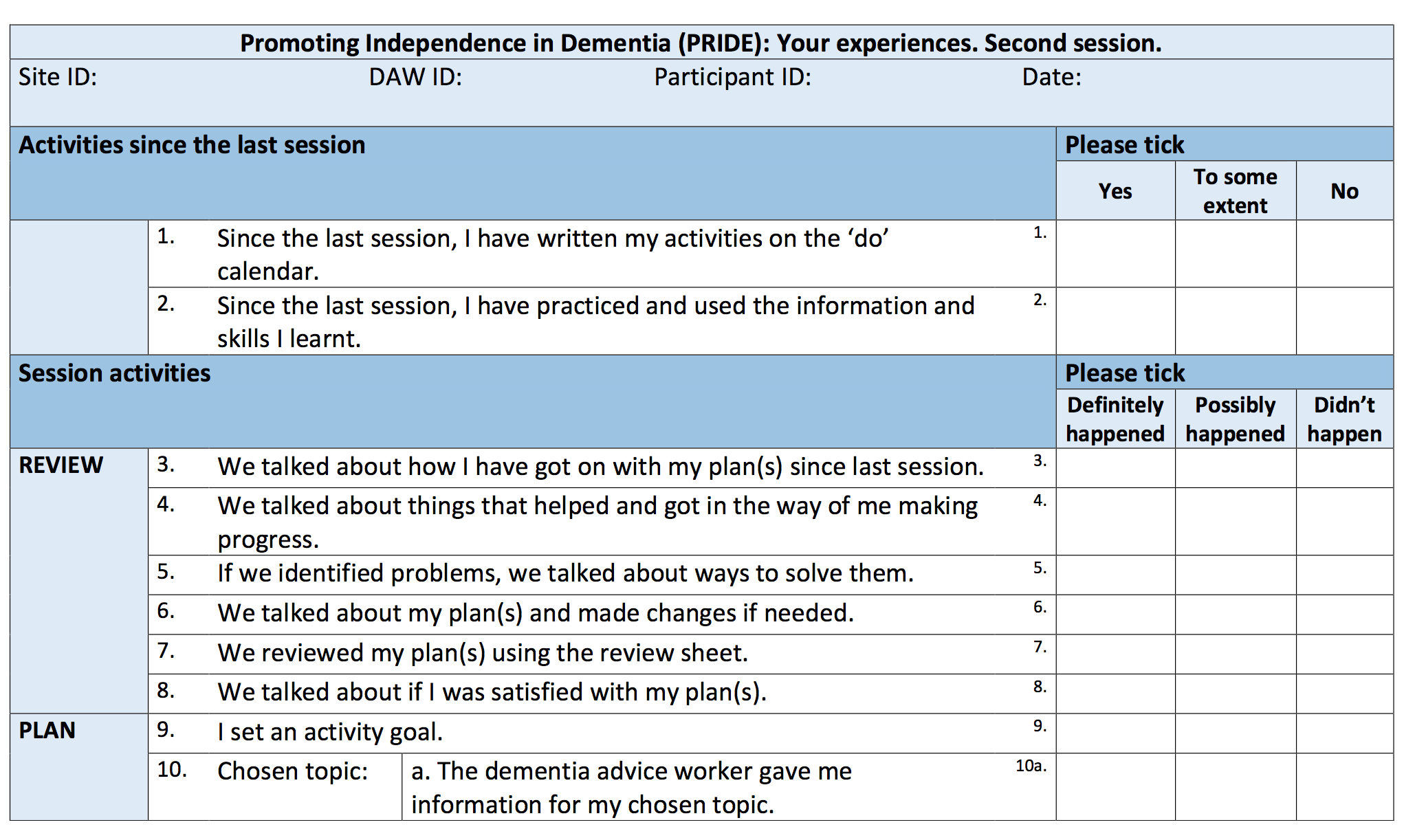


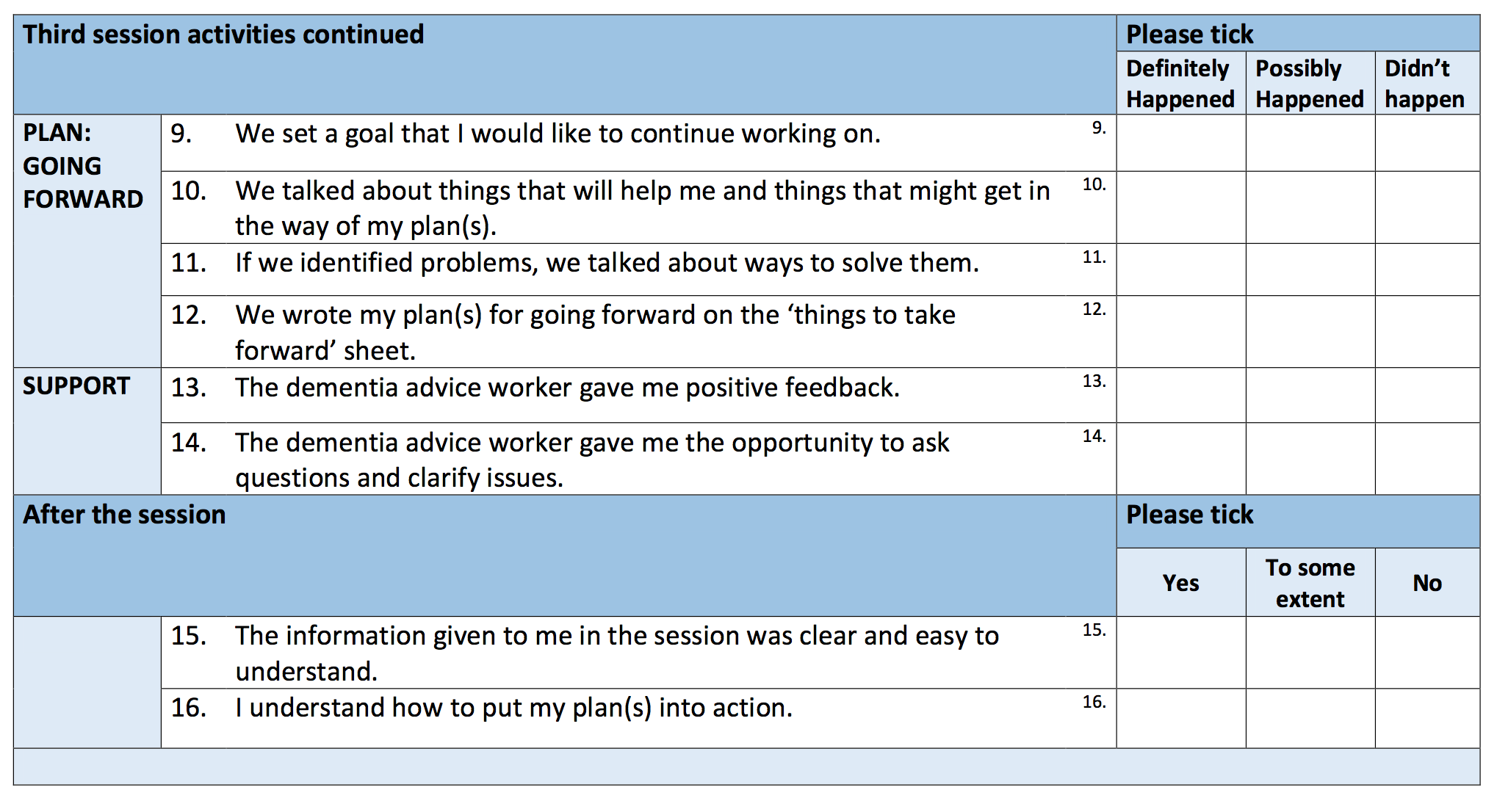

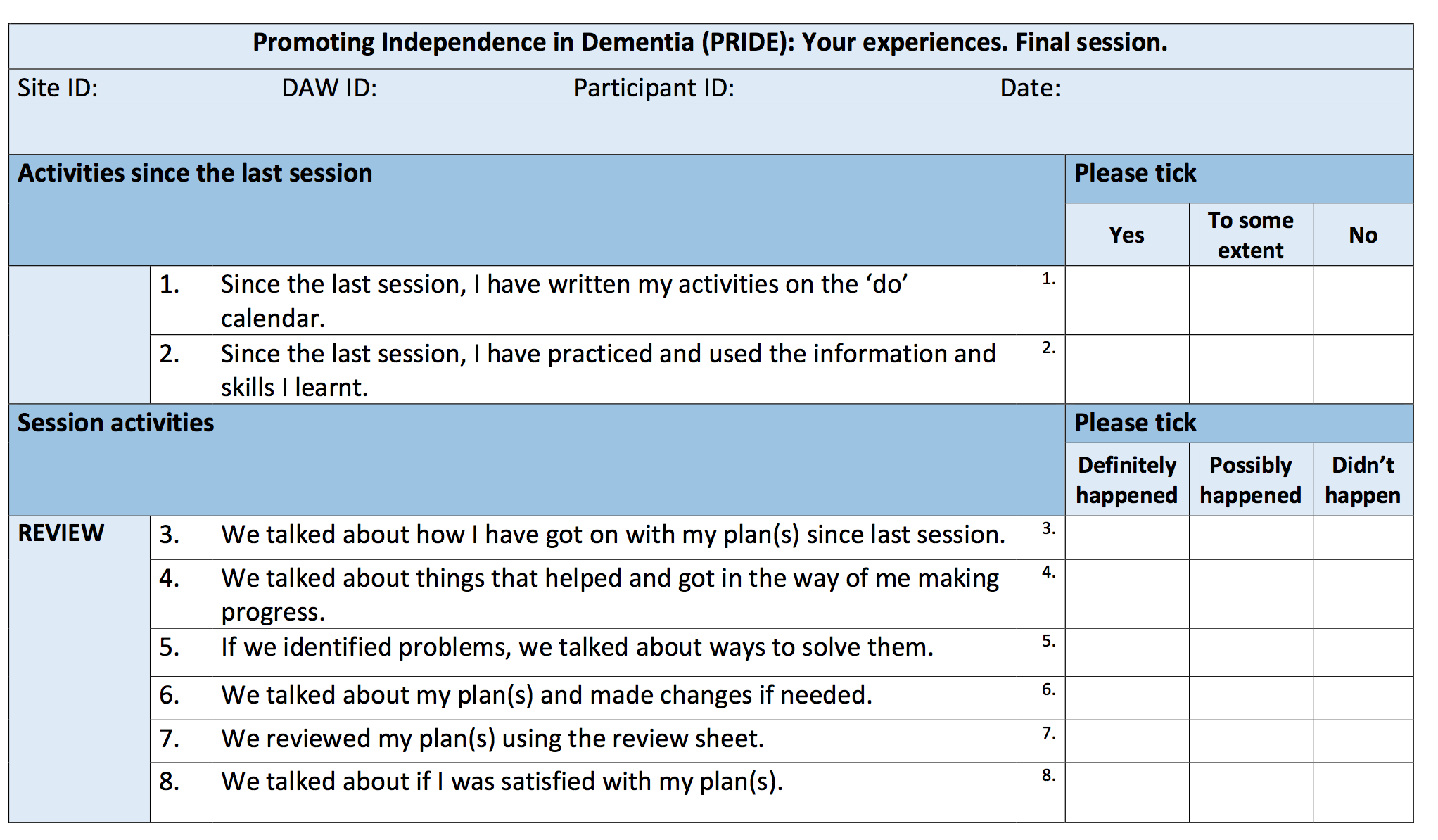

Supplement: Supplementary file 1 — Appendix S1. PRIDE Intervention framework. Appendix S2. PRIDE coding guidelines for researchers. Appendix S3. Provider/researcher fidelity checklists, Sessions 1–3. Appendix S4. Participant ‘your experience’ fidelity checklists, Sessions 1–3. [file BJHP-25-39-s001.docx]
